# Synthesis of (−)-halichonic acid and (−)-halichonic acid B

**DOI:** 10.3762/bjoc.18.174

**Published:** 2022-12-01

**Authors:** Keith P Reber, Emma L Niner

**Affiliations:** 1 Department of Chemistry, Towson University, 8000 York Road, Towson, MD, 21252, USA

**Keywords:** alkaloid, amino acid, aza-Prins reaction, cascade reaction, natural product

## Abstract

The first syntheses of the amino acids (–)-halichonic acid and (–)-halichonic acid B have been achieved in ten steps starting from commercially available (−)-α-bisabolol. The optimized synthetic route includes a new purification method for isolating (−)-7-amino-7,8-dihydrobisabolene in enantiomerically pure form via recrystallization of its benzamide derivative. The key intramolecular aza-Prins reaction forms the characteristic 3-azabicyclo[3.3.1]nonane ring system of halichonic acid along with the lactonized form of halichonic acid B in an 8:1 ratio. Optical rotation measurements confirmed that these synthetic compounds were in fact the enantiomers of the natural products, establishing both the relative and absolute configurations of the halichonic acids.

## Introduction

**Figure 1:**
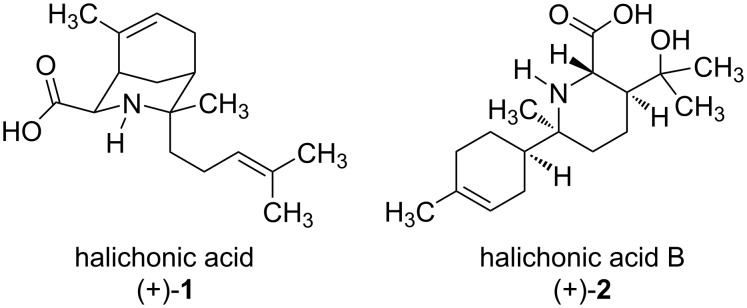
Structures of halichonic acid ((+)-**1**) and halichonic acid B ((+)-**2**).

Marine sponges produce a large number of structurally diverse natural products, including many that exhibit biological activity [1–3]. In 2019, Tsukamoto and co-workers isolated the aminobisabolene sesquiterpenoid halichonic acid ((+)-**1**) from the sponge *Halichondra* sp. (Figure 1) [4]. This amino acid natural product features a rigid 3-azabicyclo[3.3.1]nonane ring system containing four stereogenic centers within the piperidine ring. In 2021, the same group re-isolated (+)-**1** from the sponge *Axinyssa* sp. along with the structurally related compound halichonic acid B ((+)-**2**) [5]. Structurally, (+)-**2** is a pipecolic acid derivative containing a cyclohexenyl ring as a substituent group. This compound also features four stereogenic centers (three of which are located within the piperidine ring) and a tertiary alcohol. The structures of compounds (+)-**1** and (+)-**2** were elucidated through a combination of HRMS and NMR spectroscopy, while the relative configuration of each compound was established through nuclear Overhauser effect (NOE) correlations. Additionally, the absolute configuration of each compound was determined based on calculated electronic circular dichroism (ECD) spectra that were compared to the experimental ECD spectra of (+)-**1** and (+)-**2**. Although these natural products did not exhibit antimicrobial activity or cytotoxicity against HeLa cells, their biological activities in other assays have not yet been investigated.

Our group was particularly interested in the structure of halichonic acid ((+)-**1**), which shares the same bridged bicyclic ring system found in many of the *Aristotelia* alkaloids [6]. Since our lab recently reported a synthesis of the natural product aristoquinoline [7], we viewed the halichonic acids as ideal targets to extend the scope of our synthetic methodology. Given the structural similarity between compounds (+)-**1** and (+)-**2** (and the fact that they were co-isolated from the same sponge), Tsukamoto et al. proposed that these natural products could be derived from a common biosynthetic pathway starting from farnesyl pyrophosphate and glycine [5]. This prompted us to investigate a biomimetic synthesis in which the halichonic acids could be prepared from a common imine intermediate via divergent intramolecular aza-Prins cyclizations [8]. Herein, we report the first syntheses of the enantiomers of these natural products (i.e., (−)-**1** and (−)-**2**), confirming the structural assignments of the halichonic acids and establishing their absolute configurations.

## Results

**Scheme 1:**
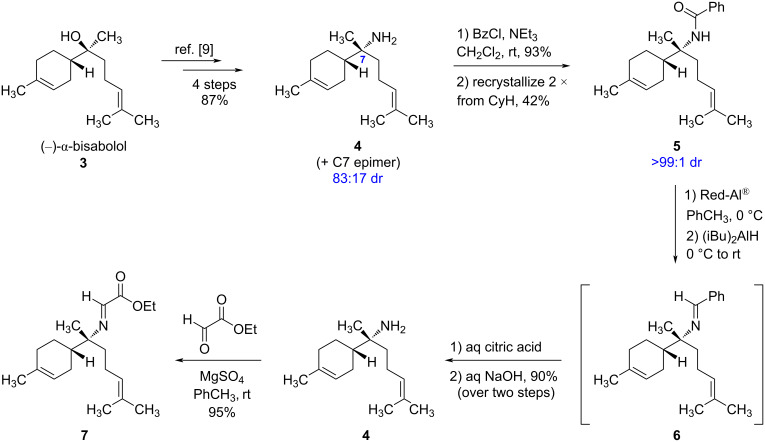
Synthesis of (−)-7-amino-7,8-dihydrobisabolene (**4**) and its conversion to cyclization precursor **7**.

Our synthetic route began with the readily available sesquiterpenoid (−)-α-bisabolol (**3**), as shown in Scheme 1. In 2013, Shenvi and co-workers reported an operationally simple and high-yielding method for converting tertiary alcohols (including **3**) to the corresponding primary amines via the intermediacy of an isonitrile [9]. This four-step procedure was conveniently carried out on a multigram scale, affording (−)-7-amino-7,8-dihydrobisabolene (**4**) and its C7-epimer as an 83:17 mixture of diastereomers in 87% overall yield. Unfortunately, these diastereomers were not separable by conventional column chromatography. Although this diastereomeric mixture could be converted into a variety of amine derivatives (e.g., hydrochloride salt, mandelic acid salt, phthalimide, ketopinic acid amide, salicylaldehyde imine, *p*-toluenesulfonamide, acetamide, etc.), all attempts to separate the resulting isomers (which were oils) were similarly unsuccessful.

Seeking an alternative to column chromatography, we decided to prepare solid derivatives of **4** (and its C7-epimer) that could be purified by recrystallization. Although the α-bromoacetamide [10] and *p*-bromobenzamide derivatives of these amines are solids, no change in dr was observed upon recrystallization from a variety of solvents. Fortunately, we ultimately found success with the benzamide derivative **5**, which could be prepared from the mixture of **4** and its C7-epimer in 93% yield upon treatment with benzoyl chloride. Recrystallization of the resulting mixture of diastereomeric amides from cyclohexane improved the dr from 83:17 to 95:5 (as determined by ^1^H NMR based on integration of the C7-methyl signals). A second recrystallization from cyclohexane afforded **5** as a single stereoisomer (>99:1 dr) with 42% overall recovery of material (corresponding to 51% recovery of the major diastereomer **5**).

Having finally separated the C7-diastereomers, we anticipated that the amide **5** could be hydrolyzed to give a single enantiomer of amine **4**. However, we found that amide **5** was remarkably resistant to hydrolysis, even under forcing conditions. For example, no amide hydrolysis was observed in concentrated aqueous NaOH solution at reflux (with or without an organic co-solvent), and slow decomposition occurred under acidic conditions at elevated temperatures. Alternative methods to cleave the benzamide using sodium peroxide [11] or triethyloxonium tetrafluoroborate [12] were also unsuccessful, giving either no reaction or significant decomposition, respectively. At this stage, we started to investigate alternative methods to cleave the amide via reduction. Achieving selective C–N-bond cleavage of amides under reductive conditions is still a largely unsolved problem since a C–O-bond cleavage is typically the preferred mode of reactivity, especially when using hydride reducing agents [13]. Nevertheless, specialized conditions for achieving C–N-bond cleavage of amides using SmI_2_ [13], Tf_2_O/Et_3_SiH [14], and stoichiometric Schwartz’s reagent [15] have been reported; however, none of these methods was successful in reducing amide **5** to the desired amine **4**.

Although there is one literature example of directly reducing a benzamide with diisobutylaluminum hydride (DIBAL) to achieve C–N-bond cleavage [16], we observed exclusive over-reduction of compound **5** under these conditions to form the corresponding *N*-benzylamine, even at −78 °C. We next investigated the reducing agent sodium bis(2-methoxyethoxy)aluminum hydride (Red-Al^®^), which is a convenient alternative to LiAlH_4_ that exhibits high solubility in organic solvents and is also known to reduce amides [17]. When a solution of amide **5** in toluene was treated with an excess of Red-Al^®^ at 0 °C, rapid gas evolution (likely H_2_) occurred. However, no reduction of the amide was observed, even after stirring at room temperature for 24 hours. In an effort to “salvage” the reaction by reducing the amide to the corresponding *N*-benzylamine (which could potentially be oxidized to the corresponding imine with IBX [18] and subsequently hydrolyzed to give **4**), we added excess DIBAL and allowed the reaction mixture to stir at room temperature for an additional 24 hours. Upon quenching the reaction with a saturated aqueous solution of potassium sodium tartrate (Rochelle’s salt), we were astonished to observe the clean formation of imine **6**. Presumably, the combination of Red-Al^®^ and DIBAL reacts with amide **5** to form a stable tetrahedral intermediate that collapses to **6** upon aqueous workup. This type of direct amide semi-reduction using aluminum hydride reagents is, to the best of our knowledge, previously unknown within the chemical literature and is especially notable since it does not require cryogenic temperatures. Efforts to further investigate the scope of this unique transformation are currently underway in our laboratory and will be reported in due course.

Attempts to purify imine **6** by column chromatography on silica gel resulted in extensive decomposition. Therefore, the crude imine was immediately hydrolyzed using aqueous citric acid [14], affording (−)-7-amino-7,8-dihydrobisabolene (**4**) as a single stereoisomer in 90% yield over the two steps. The enantiomer of **4** is itself a natural product with cytotoxic, antifungal, and antimicrobial properties [10,19–22]. Notably, (+)-**4** was also co-isolated with compounds (+)-**1** and (+)-**2** in sponge extracts, suggesting that these compounds may share a common biosynthetic pathway [4–5]. Both enantiomers of **4** have been previously synthesized [9,23–24], and this compound has also been prepared in racemic form [25]. To supply the final two carbon atoms found in the halichonic acids, amine **4** was condensed with a solution of ethyl glyoxylate in toluene, giving imine **7** in 95% yield. We found that imine **7** could be purified by column chromatography on silica gel if the mobile phase contained approximately 2% triethylamine as a basic additive. However, it is also possible to use crude **7** in the subsequent cyclization step without significantly affecting isolated yields. ^1^H NMR analysis showed that **7** was formed as a single geometrical isomer; although the imine configuration was not rigorously established, we have assigned it as the (*E*)-isomer, as is commonly observed in aldimine formation.

**Scheme 2:**
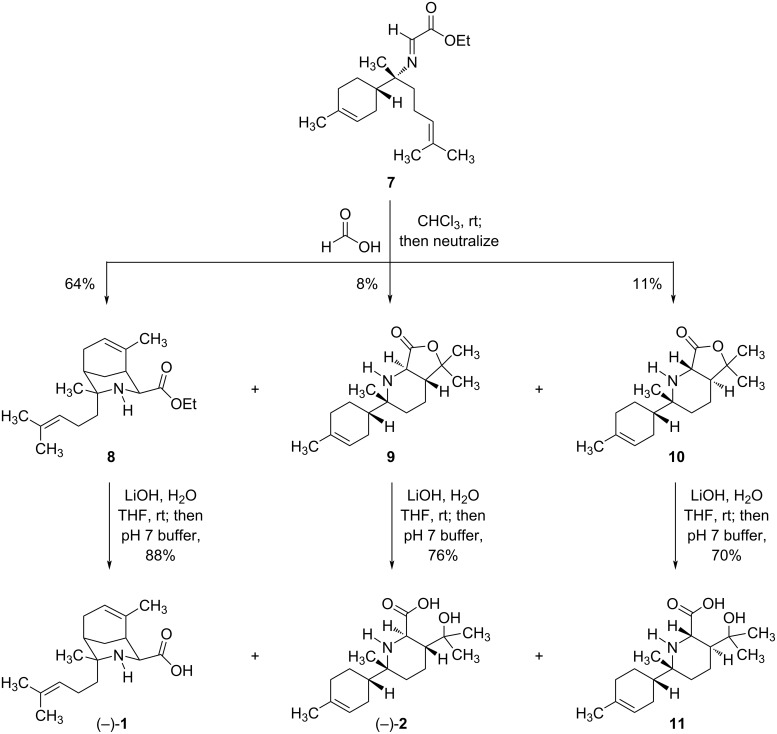
Synthesis of the halichonic acids via a key intramolecular aza-Prins cyclization.

The stage was now set for the key intramolecular aza-Prins reaction that would form the bicyclic structures of the halichonic acids (Scheme 2). When a solution of imine **7** in chloroform was treated with a large excess (85–100 equiv) of formic acid at room temperature, we were pleased to observe the formation of bicyclic compound **8** as the major product in 64% yield. Notably, **8** is the ethyl ester of (−)-halichonic acid and features the characteristic 3-azabicyclo[3.3.1]nonane ring system found in this natural product. However, we were intrigued that a competing cyclization process also formed isomeric lactones **9** and **10** in 8% yield and 11% yield, respectively. ^1^H NMR analysis confirmed that compounds **9** and **10** were both *trans*-fused 6/5 bicycles based on the magnitude of the vicinal coupling constant between the two methine hydrogens at the ring fusion (^3^*J* = 12.9 Hz). The rigid nature of the *trans*-fused 6/5 ring system results in distinct conformers for **9** and **10**; fortunately, this allowed for the unambiguous assignment of the relative configurations of these diastereomers via NMR based on nuclear Overhauser effect (NOE) correlations (see the Supporting Information File 1 for additional details). This analysis showed that the minor product **9** corresponded to the lactone of (−)-halichonic acid B.

At this point, all that remained to complete the syntheses of the halichonic acids was hydrolysis of compounds **8** and **9** to form the corresponding amino acids. Thus, treating bicycle **8** with aqueous lithium hydroxide resulted in hydrolysis of the ethyl ester, and subsequent neutralization with pH 7 phosphate buffer afforded halichonic acid ((−)-**1**) in 88% yield after purification by column chromatography. Similarly, hydrolysis of lactone **9** under analogous conditions afforded halichonic acid B ((−)-**2**) in 76% yield. ^1^H and ^13^C NMR data for the synthetic compounds (−)-**1** and (−)-**2** were identical to those reported for the halichonic acids, confirming the proposed structures of these natural products. However, the observed optical rotations of these synthetic compounds were of opposite sign to those reported for the halichonic acids. Since we synthesized the enantiomers of these natural products, the absolute configurations of (+)-**1** and (+)-**2** assigned by Tsukamoto et al. have now been experimentally confirmed [4–5]. For the sake of comparison, the diastereomeric lactone **10** was also hydrolyzed under the same conditions to form the “unnatural” product **11** in 70% yield, which we have designated (−)-isohalichonic acid B. Although the NMR spectra of (−)-**2** and **11** are quite similar, we did note a significant difference in the ^13^C NMR chemical shift of the C7-methyl group, which appears at δ = 20.7 in (−)-**2** and δ = 14.5 in **11**.

## Discussion

**Scheme 3:**
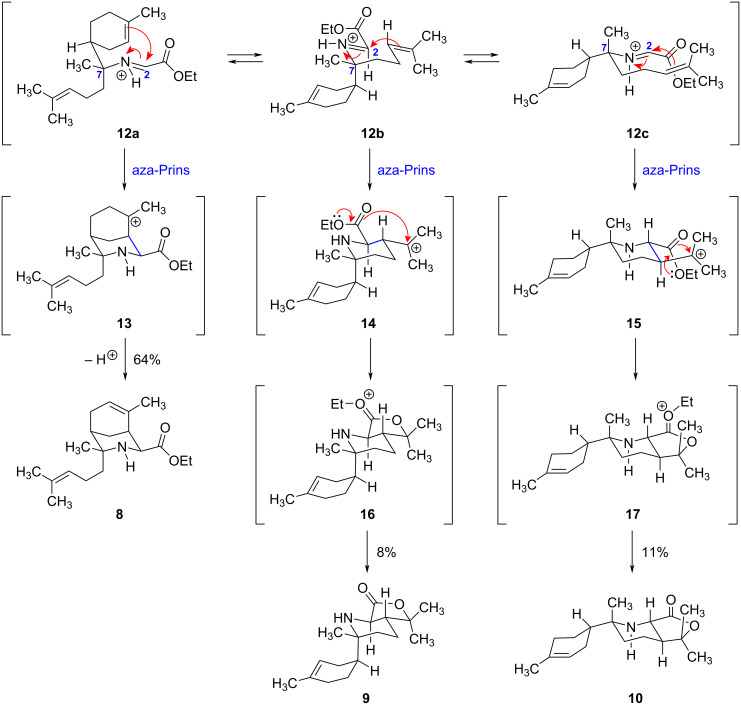
Proposed intermediates for the intramolecular aza-Prins reaction leading to the formation of ethyl ester **8** and isomeric lactones **9** and **10**.

Rationalizing the outcome of the aza-Prins reaction leading to the formation of ethyl ester **8** and isomeric lactones **9** and **10** (Scheme 2) provides an interesting exercise in acyclic conformational analysis. Three divergent mechanistic pathways can be formulated by considering the different conformers of protonated imine **7**, namely iminium ions **12a**–**c** (Scheme 3). In each case, the chair-like transition state of the intramolecular aza-Prins reaction is controlled by the C7-stereogenic center, which bears a methyl group, the electrophilic site (the iminium ion), and two possible nucleophilic sites (a prenyl group and a trisubstituted alkene within a cyclohexene ring).

In conformer **12a**, the prenyl group occupies a pseudo-axial position, the methyl group occupies a pseudo-equatorial position, and the trisubstituted alkene within the six-membered ring serves as the nucleophile. It is important to note that in this chair-like conformer, the ethyl ester group at C2 assumes a pseudo-equatorial position. Although an alternative boat-like conformer is also possible (which would ultimately lead to the C2-epimer of **8**), the resulting transition state is presumably much higher in energy. In practice, the intramolecular aza-Prins reaction of **12a** forms a new carbon–carbon bond to generate a rigid 3-azabicyclo[3.3.1]nonane ring system (**13**). Although several different fates could be envisioned for this carbocation (e.g., a nucleophilic attack of formic acid to give a formate ester), only alkene formation was observed in this system. Interestingly, the deprotonation step is completely regioselective, giving the more highly substituted endocyclic trisubstituted alkene found in **8** as opposed to the isomeric exocyclic 1,1-disubstituted alkene [7–8]. Alternative mechanistic pathways involving (1) deprotonation to form a bridgehead alkene, or (2) intramolecular nucleophilic attack of the ethyl ester to form a lactone are not possible in this system due to the rigid geometric constraints of the 3-azabicyclo[3.3.1]nonane ring system. In any case, this aza-Prins reaction is by far the preferred mode of cyclization of iminium ion **12** based on the isolated yield of **8** (64%), ultimately leading to the carbon skeleton found in the natural product halichonic acid ((+)-**1**).

In conformer **12b**, the cyclohexenyl ring system occupies a pseudo-axial position, the methyl group occupies a pseudo-equatorial position, and the trisubstituted alkene of the prenyl group serves as the nucleophile. The chair-like transition state of the intramolecular aza-Prins reaction allows both the ethyl ester and the resulting tertiary carbocation to occupy equatorial positions (**14**), establishing the observed *trans*-relationship between these groups while simultaneously setting two new stereogenic centers. As before, one can envision several different fates for the tertiary carbocation present in **14**. Although elimination to form an alkene or intermolecular nucleophilic attack by formic acid (ultimately giving a formate ester) are reasonable mechanistically, only the intramolecular nucleophilic attack by the carbonyl group of the pendent ethyl ester was observed in this system to form the resonance-stabilized oxocarbenium ion **16**. Subsequent loss of the ethyl group (either as ethyl formate upon solvolysis with the formic acid co-solvent or as ethanol upon aqueous workup) gives lactone **9**, which features a strained *trans*-fused 6/5 ring system. Although this lactone survives aqueous workup at neutral pH, it is rapidly hydrolyzed under basic conditions (Scheme 2) to form the enantiomer of halichonic acid B ((−)-**2**). It is interesting to note that halichonic acid B exists exclusively as an open-chain 4-hydroxycarboxylic acid even though the corresponding γ-lactones typically form spontaneously. Indeed, no lactone formation was observed from (−)-**2** even upon purification by column chromatography on silica gel, reflecting the highly strained nature of *trans*-fused lactone **9**.

Finally, conformer **12c** is similar to **12b** in that the trisubstituted alkene of the prenyl group once again serves as the nucleophile; however, the methyl group now occupies a pseudo-axial position, and the cyclohexenyl ring system occupies a pseudo-equatorial position. In this case, the aza-Prins reaction forms *trans*-fused lactone **10** via an analogous intramolecular nucleophilic attack of the ethyl ester on the intermediate tertiary carbocation **15** to give oxocarbenium ion **17**. In comparing conformers **12b** and **12c**, it appears that the chair-like transition state **12c** should be lower in energy since the more sterically demanding cyclohexenyl ring is located in a pseudo-equatorial position. Although we did observe a slightly higher yield of **10** as compared to **9** (11% vs 8%), these values are sufficiently close to make any conclusions regarding the effects of conformational preferences on reactivity tenuous at best. However, it is interesting to note that the enantiomer of **11** has not been co-isolated as a natural product along with compounds (+)-**1** and (+)-**2**. If the biosyntheses of these natural products does occur through a common iminium ion intermediate, then our isolation of **11** suggests that the key aza-Prins cyclization is enzyme-mediated rather than spontaneous.

## Conclusion

In summary, we have synthesized the enantiomers of halichonic acid and halichonic acid B in 10 steps starting from commercially available (−)-α-bisabolol (**3**). An important intermediate in our route was (−)-7-amino-7,8-dihydrobisabolene (**4**), which was prepared in enantiomerically pure form following recrystallization of a diastereomeric mixture of the corresponding benzamides. A common imine intermediate (**7**) underwent two different intramolecular aza-Prins reactions in the presence of formic acid to give the ethyl ester of (−)-**1** and the lactone of (−)-**2** in 64% yield and 8% yield, respectively. Subsequent hydrolysis of these intermediates under basic conditions afforded (−)-halichonic acid and (−)-halichonic acid B, confirming the proposed structures of the natural products. Efforts to investigate the biological activities of compounds (−)-**1** and (−)-**2** and their synthetic analogs are currently underway in our laboratory.

## Supporting Information

The supporting information file contains detailed experimental procedures, full characterization data and copies of ^1^H and ^13^C NMR spectra for all new compounds, and complete NMR spectral assignments for compound (−)-**1**, (−)-**2**, **8**, **9**, **10**, and **11**. A tabular comparison between the NMR data reported for natural products (+)-**1** and (+)-**2** and that obtained for their synthetic enantiomers (−)-**1** and (−)-**2** is also provided.

File 1 Experimental procedures, characterization data and copies of ^1^H and ^13^C NMR spectra.
