# Supplementary material for: Synthesis of (−)-halichonic acid and (−)-halichonic acid B
Source: Beilstein J Org Chem. 2022 Dec 1;18:1629–35. doi: 10.3762/bjoc.18.174 (PMC9727271; doi:10.3762/bjoc.18.174)
Supplement: File 1 — Experimental procedures, characterization data and copies of 1H and 13C NMR spectra. [file Beilstein_J_Org_Chem-18-1629-s001.pdf]

BEILSTEIN JOURNAL OF ORGANIC CHEMISTRY

Supporting Information

for

Synthesis of (−)-halichonic acid and (−)-halichonic acid B

Keith P. Reber and Emma L. Niner

Beilstein J. Org. Chem. **2022,** *18,* 1629–1635. doi:10.3762/bjoc.18.174

Experimental procedures, characterization data and copies of ^{1}H and ^{13}C NMR spectra

License and Terms: This is a supporting information file under the terms of the Creative Commons Attribution License (https://creativecommons.org/licenses/by/4.0). Please note that the reuse, redistribution and reproduction in particular requires that the author(s) and source are credited and that individual graphics may be subject to special legal provisions.
The license is subject to the *Beilstein Journal of Organic Chemistry* terms and conditions: (https://www.beilstein-journals.org/bjoc/terms)

Table of contents

Synthetic procedures

(−)-7-Amino-7,8-dihydro-α-bisabolene (**4**) and its C7 epimer S2–S3

Benzamide **5** S4

(−)-7-Amino-7,8-dihydro-α-bisabolene (**4**) S5–S6

Imine **7** S7

(−)-Halichonic acid ethyl ester (**8**) and lactones **9** and **10** S8–S9

(−)-Halichonic acid [(−)-**1**] S10

(−)-Halichonic acid B [(−)-**2**] S11

(−)-Isohalichonic acid B (**11**) S12

^{1}H NMR and ^{13}C NMR spectra

Benzamide **5** S13–S14

(−)-7-Amino-7,8-dihydro-α-bisabolene (**4**) S15–S16

Imine **7** S17–S18

(−)-Halichonic acid ethyl ester (**8**) S19–S20

Lactone **9** S21–S22

Lactone **10** S23–S24

(−)-Halichonic acid [(−)-**1**] S25–S26

(−)-Halichonic acid B [(−)-**2**] S27–S28

(−)-Isohalichonic acid B (**11**) S29–S30

Complete ^{1}H NMR and ^{13}C NMR assignments

(−)-Halichonic acid ethyl ester (**8**) S31

Lactone **9** S32

Lactone **10** S33

(−)-Halichonic acid [(−)-**1**] S34

(−)-Halichonic acid B [(−)-**2**] S35

(−)-Isohalichonic acid B (**11**) S36

References S37

Synthesis of (−)-7-amino-7,8-dihydro-α-bisabolene (4) and its C7 epimer:

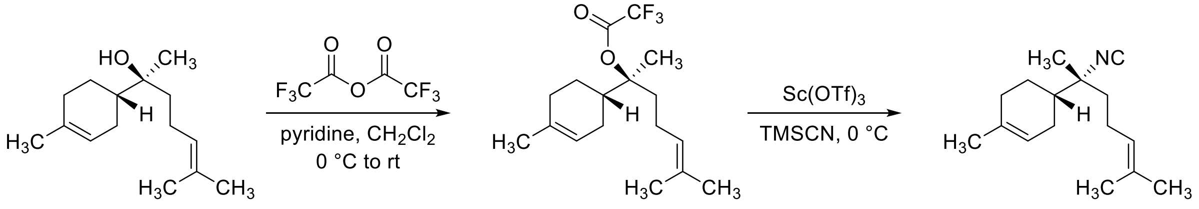

(–)-α-bisabolol
3

SI-1

SI-2
83:17 dr

This four-step procedure for the synthesis of (−)-7-amino-7,8-dihydro-α-bisabolene (**4**) and its C7-epimer is adapted from the one originally reported by Shenvi et al.[1]

To a flame-dried 200 mL flask under argon equipped with a magnetic stirring bar was added a solution of (−)-α-bisabolol (**3**, 2.00 g, 8.99 mmol, 1.00 equiv) in 43 mL of anhydrous CH_2Cl_2. This colorless solution was cooled to 0 °C in an ice bath, and neat pyridine (2.88 mL, 35.6 mmol, 4.00 equiv) was added followed by the dropwise addition of neat trifluoroacetic anhydride (2.53 mL, 18.2 mmol, 2.02 equiv). After stirring at 0 °C for 1.5 hours, the reaction was quenched with 1 M aq HCl solution, and the resulting biphasic mixture was stirred vigorously at room temperature for five minutes. The layers were separated, and the aqueous phase was extracted with one additional portion of CH_2Cl_2 before the combined organic layers were dried over anhydrous Na_2SO_4. TLC (3:1 hexanes/EtOAc, anisaldehyde) showed complete consumption of the (−)-α-bisabolol (R_f = 0.46, stains blue) and clean formation of the trifluoroacetate ester (R_f = 0.74, stains blue). The drying agent was removed by vacuum filtration, and the solvent was evaporated under reduced pressure to afford the crude trifluoroacetate ester **SI-1** (2.8630 g) as a light yellow oil that was used directly in the next step.

To a flame-dried 50 mL flask under argon containing neat trifluoroacetate ester **SI-1** (2.863 g, 8.99 mmol, 1.00 equiv) and a magnetic stirring bar was added neat trimethylsilyl cyanide (9.50 mL, 75.9 mmol, 8.44 equiv), and the resulting pale yellow solution was maintained at 0 °C in an immersion cooler. In a separate flame-dried 10 mL flask, solid scandium(III) triflate (132.7 mg, 0.270 mmol, 0.03 equiv) was briefly heated with a heat gun under high vacuum and then allowed to cool to room temperature. The dried scandium(III) triflate was then dissolved in trimethylsilyl cyanide (4.00 mL, 32.0 mmol, 3.56 equiv), and the resulting colorless solution was added dropwise to the cooled flask containing the trifluoroacetate ester. The reaction mixture was stirred at 0 °C for 24 hours, at which point it had turned dark orange/red. After warming to room temperature, the reaction mixture was quenched via portionwise addition (≈ 1 mL at a time using a Pasteur pipette) to a vigorously stirred biphasic mixture of Et_2O (100 mL) and 1 M aq NaOH (100 mL) contained in a 500 mL Erlenmeyer flask. The resulting biphasic mixture was stirred vigorously for five minutes before the layers were separated. The aqueous phase was extracted with one additional portion of Et_2O before the combined organic layers were dried over

anhydrous $MgSO_4$. TLC (3:1 hexanes/EtOAc, anisaldehyde) showed complete consumption of the trifluoroacetate ester (R_f = 0.74, stains blue) and formation of the isonitrile (R_f = 0.65, stains green). The drying agent was removed by vacuum filtration, the solvent was evaporated under reduced pressure, and the residue was dried under high vacuum (to remove trimethylsilanol) to give the crude isonitrile **SI-2** (2.08 g) as a dark orange oil. ^{1}H NMR analysis showed that the isonitrile had been formed as an 83:17 mixture of diastereomers, favoring inversion at the tertiary center undergoing substitution. This material was used directly in the subsequent hydrolysis step without purification.

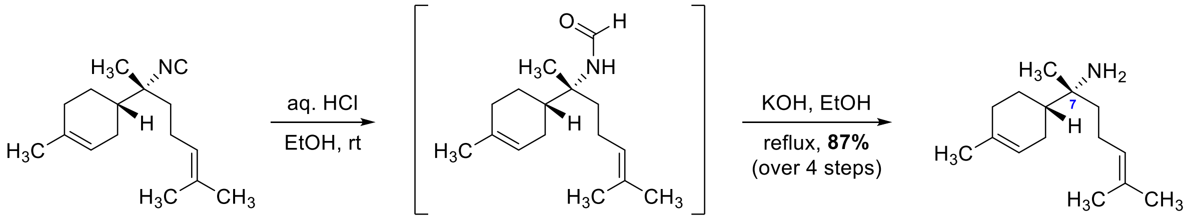

SI-2
83:17 dr

SI-3

4
(+ C7 epimer)
83:17 dr

To a 200 mL flask under argon equipped with a magnetic stirring bar was added a solution of crude isonitrile **SI-2** (2.08 g, 8.99 mmol, 1.00 equiv) in 90 mL of 95% ethanol. A 3 M aqueous solution of HCl (4.50 mL, 13.5 mmol, 1.50 equiv) was added, and the reaction was stirred at room temperature for 5 hours. After this time, TLC (3:1 hexanes/EtOAC, anisaldehyde) showed complete consumption of the isonitrile (R_f = 0.65, stains green) and formation of the corresponding formamide **SI-3** (R_f = 0.15, stains blue) along with a small amount of the amine (R_f = 0, stains blue). Solid potassium hydroxide (2.90 g, 51.7 mmol, 5.75 equiv) was added, the flask was equipped with a water-cooled condenser, and the reaction mixture was heated at reflux for 20 hours. After cooling to room temperature, the reaction mixture was concentrated under reduced pressure (to remove the ethanol) and then partitioned between water and hexanes. The layers were separated, and the aqueous phase was extracted with two additional portions of hexanes before the combined organics layers were dried over anhydrous Na_2CO_3. TLC (9:1 CH_2Cl_2/CH_3OH, anisaldehyde) of the organic phase showed clean formation of the product as a streaky spot of R_f = 0.22 (stains blue). The drying agent was removed by vacuum filtration, and the solvent was evaporated under reduced pressure to give the crude amine as a dark orange oil. This material was purified by column chromatography (9:1 CH_2Cl_2/CH_3OH) to afford a mixture of amine **4** and its C7 epimer (1.7316 g, 87%) as a yellow oil.

Spectroscopic data for major diastereomer **4** were consistent with that reported by Shenvi et al.[1] In particular, ^{1}H NMR analysis showed that the amine was also an 83:17 mixture of diastereomers, matching the dr of the isonitrile.

Synthesis of diastereomerically pure benzamide 5:

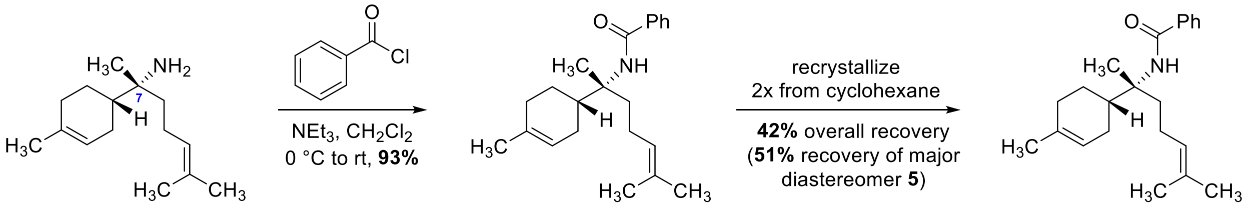

To a flame-dried 250 mL flask under argon equipped with a magnetic stirring bar was added a solution of amine **4** and its C7 epimer (2.6150 g, 11.8 mmol, 1.00 equiv, 83:17 mixture of diastereomers) in 100 mL of anhydrous CH_2Cl_2. This solution was cooled to 0 °C in an ice bath, and neat triethylamine (2.06 mL, 14.8 mmol, 1.25 equiv) was added followed by the dropwise addition of neat benzoyl chloride (1.48 mL, 12.8 mmol, 1.08 equiv). The cooling bath was allowed to slowly warm to room temperature, and the reaction was stirred for 15 hours. After this time, the reaction was quenched with water, and the layers were separated. The organic phase was sequentially washed with 1 M aq HCl solution and 1 M aq NaOH before drying over anhydrous sodium sulfate. TLC (3:1 hexanes/EtOAc, UV/anisaldehyde) showed complete consumption of the amine (R_f = 0.10, not UV-active) and clean formation of the amide **5** (R_f = 0.56, UV-active, stains blue). The drying agent was removed by filtration, and the solvent was evaporated under reduced pressure to give the crude product as a yellow solid. This material was purified by column chromatography (100% CH_2Cl_2) to afford a mixture of amide **5** and its C7 epimer (3.5702 g, 93%) as a white solid. ^{1}H NMR showed that this material was still an 83:17 mixture of diastereomers (based on integration of the CH_3 singlets at 1.33 ppm and 1.37 ppm, respectively), matching the dr of the starting amine. This mixture of diastereomeric amides was recrystallized from 70 mL of cyclohexane to give 1.8194 g of material that was enriched to 95:5 dr. A second recrystallization from 25 mL of cyclohexane afforded 1.5172 g of diastereomerically pure amide **5** (51% recovery of the major diastereomer; 42% overall recovery of material based on two recrystallizations).

mp 97–98 °C (cyclohexane); **$[\alpha]^{20}_D$** –20.3° (*c* 0.70, $CHCl_3$); **IR** (solid ATR): 3308, 3058, 2962, 2923, 1640, 1580, 1534, 1489, 1447, 1288, 713, 693 cm^{-1}; **^{1}H NMR** ($CDCl_3$, 400 MHz): δ = 7.70 (2H, d, *J* = 7.3 Hz), 7.46 (1H, t, *J* = 7.3 Hz), 7.40 (2H, *t*, *J* = 7.3 Hz), 5.84 (1H, br s, NH), 5.36 (1H, m), 5.15 (1H, m), 2.38 (1H, m), 2.18-1.71 (9H, m), 1.64 (3H, s), 1.63 (3H, s), 1.58 (3H, s), 1.33 (3H, s), 1.30–1.24 (1H, m); **^{13}C NMR** ($CDCl_3$, 100 MHz): δ = 166.9, 136.2, 134.2, 131.8, 131.1, 128.6, 126.7, 124.5, 120.6, 59.3, 40.5, 36.0, 31.3, 26.5, 25.8, 24.3, 23.4, 22.7, 20.9, 17.7; **HRMS** (ESI+): *m*/*z* [M+H]$^+$ calcd for $C_{22}H_{32}NO^+$: 326.2478; found: 326.2512.

Synthesis of (−)-7-amino-7,8-dihydro-α-bisabolene (4):

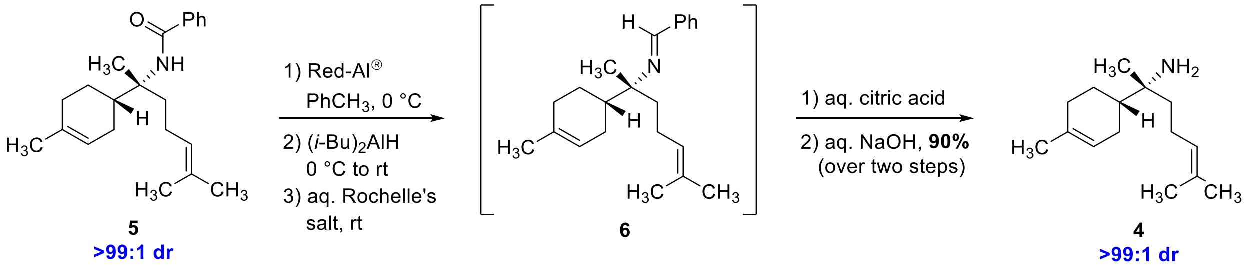

To a flame-dried 200 mL flask under argon equipped with a magnetic stirring bar was added a solution of amide **5** (983.5 mg, 3.02 mmol, 1.00 equiv) in 40 mL of anhydrous toluene. This colorless solution was cooled to 0 °C in an ice bath, and a 3.5 M solution of sodium bis(2-methoxyethoxy)aluminum hydride in toluene (Red-Al®, 2.59 mL, 9.06 mmol, 3.00 equiv) was added dropwise. Vigorous gas evolution was observed, and the resulting clear, colorless solution was stirred at 0 °C for 20 minutes. After this time, a 25% solution of diisobutylaluminum hydride in hexanes (7.36 mL, 9.06 mmol, 3.00 equiv) was added dropwise, and the solution turned cloudy. The cooling bath was allowed to slowly expire, and the reaction mixture was stirred at room temperature for 24 hours. The reaction was quenched at room temperature by the slow, dropwise addition of saturated aqueous potassium sodium tartrate solution until gas evolution ceased (≈ 5 mL). An additional 45 mL of saturated aqueous potassium sodium tartrate solution was added along with 50 mL of Et_2O, and the resulting biphasic mixture was stirred vigorously for 20 minutes at which point two clear, colorless layers were observed. The layers were separated, and the aqueous phase was extracted with one additional portion of Et_2O before the combined organic layers were dried over anhydrous $MgSO_4$. TLC (3:1 hexanes/EtOAc) showed complete consumption of the starting amide **5** (R_f = 0.56) and clean formation of imine **6** (R_f = 0.78, UV-active, stains blue). The drying agent was removed by filtration, and the solvent was removed under reduced pressure to give ≈ 1.00 g of crude imine **6** as a colorless oil that was used directly in the next step without purification.

To a 50 mL flask under argon equipped with a magnetic stirring bar was added a solution of crude imine **6** (3.02 mmol, 1.00 equiv) in 12 mL of THF. A 1 M aqueous solution of citric acid (12.08 mL, 12.1 mmol, 4.00 equiv) was then added, and the resulting colorless, homogeneous solution was stirred at room temperature for 24 hours.[2] After this time, the reaction mixture was diluted with water and Et_2O, and the two layers were separated. The aqueous phase was extracted with one additional portion of Et_2O (25 mL), and the combined organic phases (organic extract 1) were dried over anhydrous $MgSO_4$. The aqueous phase was then adjusted to pH 14 with 1 M aq NaOH solution and extracted with three 25 mL portions of Et_2O, which were combined and dried over anhydrous Na_2CO_3 (organic extract 2). TLC (3:1 hexanes/EtOAc) of organic extract 1 showed complete consumption of imine **6** (R_f = 0.78), the formation of benzaldehyde (R_f = 0.47, UV-active, does not stain), a small amount of the conjugate acid of the *N*-benzylamine overreduction product (R_f = 0.54, weakly UV-active, stains light blue), and a small amount of the conjugate acid of the

desired primary amine **4** on the baseline (R_f =0, stains dark blue). TLC (9:1 CH_2Cl_2/CH_3OH, UV/anisaldehyde) of organic extract 2 showed complete consumption of imine **6** (R_f = 0.92, stains blue, UV-active), formation of amine **4** (R_f = 0.21, stains dark blue, not UV-active) and a small amount of the *N*-benzylamine overreduction product (R_f = 0.74, weakly UV-active, stains light blue). The drying agent was removed from organic extract 2 using vacuum filtration, and the solvent was removed under reduced pressure. The crude amine was then purified by column chromatography (9:1 CH_2Cl_2/CH_3OH) to give diastereomerically pure amine **4** as a colorless oil (602.4 mg, 90% over two steps).

$[\alpha]^{20}_D$ –72.8° (*c* 0.35, $CHCl_3$) [lit. $[\alpha]^{20}_D$ –56.3° (*c* 3.2, $CHCl_3$)][3]; **IR** (thin film): 3375, 2963, 2920, 2855, 1618, 1520, 1438, 1375, 1155, 914, 798 cm^{-1}; **^{1}H NMR** ($CDCl_3$, 400 MHz): δ = 5.37 (1H, m), 5.09 (1H, m), 2.06–1.75 (7H, m), 1.67 (3H, s), 1.63 (3H, s), 1.60 (3H, s), 1.57–1.47 (1H, m), 1.46–1.39 (2H, m), 1.25 (1H, ddd, *J* = 12.1 Hz, 12.1 Hz, 5.9 Hz), 1.07 (3H, s); **^{13}C NMR** ($CDCl_3$, 100 MHz): δ = 134.0, 131.6, 124.6, 120.8, 54.4, 42.7, 39.9, 31.3, 26.2, 25.8, 24.7, 24.0, 23.4, 22.4, 17.8; **HRMS** (ESI+): *m*/*z* $[M+H]^+$ calcd for $C_{15}H_{28}N^+$: 222.2216; found: 222.2246.

Synthesis of imine 7:

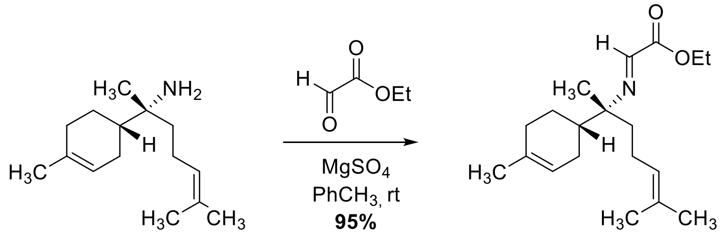

To a flame-dried 100 mL flask under argon equipped with a magnetic stirring bar was added a solution of purified amine **4** (806.4 mg, 3.64 mmol, 1.00 equiv) in 30 mL of anhydrous toluene. Solid anhydrous magnesium sulfate (1.35 g) was added in a single portion followed by the dropwise addition of a 50% solution of ethyl glyoxylate in toluene (1.08 mL, 5.46 mmol, 1.50 equiv). The reaction mixture was stirred at room temperature for 36 hours, at which point TLC (60:20:1 hexanes/EtOAc/NEt_3, UV/anisaldehyde; TLC plates pre-treated by brief immersion in 5% NEt_3 in hexanes before allowing the solvent to evaporate) showed some unreacted amine **4** (R_f = 0.19, not UV-active, stains dark blue) and formation of imine **7** (R_f = 0.63, UV-active, stains blue). A second portion of the 50% solution of ethyl glyoxylate in toluene (0.500 mL, 2.53 mmol, 0.69 equiv) was added, and the reaction mixture was stirred at room temperature for a further 24 hours. The reaction mixture was filtered to remove the drying agent, and the filtrate was evaporated under reduced pressure to give the crude imine as a light yellow oil. This material was purified by column chromatography (30:10:1 hexanes/EtOAc/NEt_3) to give imine **7** as a colorless oil (1.0574 g, 95%). ^{1}H NMR showed that imine **7** was formed exclusively as the (*E*)-geometrical isomer. Note: The crude imine can also be used directly in the subsequent aza-Prins reaction without a significant reduction in yield of the cyclization products.

[α]$^{20}_D$ –74.3° (*c* 0.82, $CHCl_3$); **IR** (thin film): 2965, 2922, 2854, 1752, 1720, 1654, 1448, 1376, 1343, 1286, 1193, 1094, 1037, 953, 800 cm^{-1}; **^{1}H NMR** ($CDCl_3$, 400 MHz): δ = 7.53 (1H, s), 5.30 (1H, m), 5.04 (1H, m), 4.32 (2H, q, *J* = 7.1 Hz), 2.01–1.66 (9H, m), 1.64 (3H, s), 1.63–1.61 (1H, m), 1.60 (3H, s), 1.54 (3H, s), 1.33 (3H, t, *J* = 7.1 Hz), 1.17 (1H, ddd, *J* = 12.1 Hz, 12.1 Hz, 5.9 Hz), 1.09 (3H, s); **^{13}C NMR** ($CDCl_3$, 100 MHz): δ = 163.7, 149.4, 133.7, 131.6, 124.3, 120.8, 66.2, 61.6, 42.8, 39.0, 31.2, 26.4, 25.8, 24.1, 23.4, 22.3, 18.0, 17.7, 14.3; **HRMS** (ESI+): *m*/*z* [M+H]$^+$ calcd for $C_{19}H_{32}NO_2^+$: 306.2428; found: 306.2474.

Synthesis of 8, 9, and 10 via an intramolecular aza-Prins reaction:

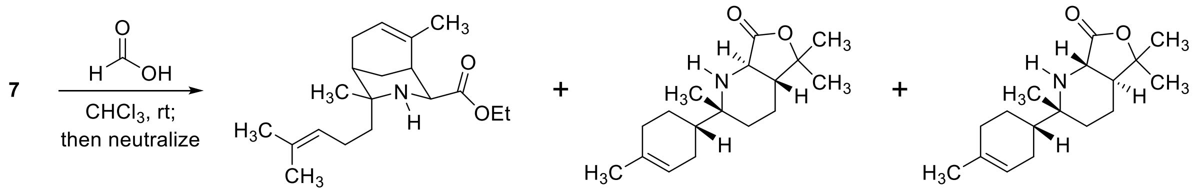

8	9	10
64%	8%	11%

To a flame-dried 100 mL flask under argon equipped with a magnetic stirring bar was added a solution of imine **7** (1.0574 g, 3.46 mmol, 1.00 equiv) in 15 mL of $CHCl_3$. Neat formic acid (10.94 mL, 294 mmol, 85.0 equiv) was added in a single portion, and the resulting light yellow homogeneous solution was stirred at room temperature for 24 hours. After this time, the acid was quenched by adding the reaction mixture portion-wise (≈ 1 mL aliquots) to a vigorously stirred biphasic mixture of CH_2Cl_2 and saturated aqueous K_2CO_3 solution contained in a 500 mL Erlenmeyer flask (gas evolution). The layers were separated, and the aqueous phase was extracted with one additional portion of CH_2Cl_2 before the combined organic layers were dried over anhydrous Na_2CO_3. TLC (3:1 hexanes/EtOAc, anisaldehyde) showed formation of halichonic acid ethyl ester **8** (R_f = 0.39), halichonic acid B lactone **9** (R_f = 0.13), and the isomeric lactone **10** (R_f = 0.26). In the alternative mobile phase 9:1 CH_2Cl_2/EtOAc, **8** appeared at R_f = 0.32, and lactone **10** appeared at R_f = 0.48. The drying agent was removed by filtration, and the solvent was removed under reduced pressure to give the crude product mixture as a yellow oil. This material was purified by column chromatography (3:1 hexanes/EtOAc) to give halichonic acid B lactone **9** (80.2 mg, 8%), some pure fractions of **8**, and several mixed fractions containing both **8** and **10**. These mixed fractions were purified by column chromatography a second time (9:1 CH_2Cl_2/EtOAc), ultimately affording pure halichonic acid ethyl ester **8** (680.4 mg, 64%) and lactone **10** (104.3 mg, 11%).

Halichonic acid ethyl ester (8):

$[\alpha]^{20}_D$ –56.4° (*c* 0.26, $CHCl_3$); **IR** (thin film): 2970, 2923, 1742, 1448, 1375, 1340, 1315, 1292, 1260, 1197, 1178, 1137, 1121, 1092, 1059, 1034, 864, 807, 736 cm^{-1}; **^{1}H NMR** ($CDCl_3$, 400 MHz): δ = 5.62 (1H, m), 5.10 (1H, m), 4.19 (1H, dq, *J* = 10.8 Hz, 7.2 Hz), 4.07 (1H, dq, *J* = 10.8 Hz, 7.2 Hz), 3.75 (1H, d, *J* = 2.8 Hz), 2.43 (1H, m), 2.20 (1H, m), 2.11 (2H, m), 2.00–1.84 (2H, m), 1.67 (3H, d, *J* = 0.9 Hz), 1.59 (3H, s), 1.57–1.54 (2H, m), 1.50 (1H, m), 1.62 (1H, m), 1.49 (3H, m), 1.27 (3H, t, *J* = 7.2 Hz), 1.08 (3H, s); **^{13}C NMR** ($CDCl_3$, 100 MHz): δ = 173.5, 131.8, 131.5, 125.6, 124.9, 60.7, 56.3, 55.1, 37.7, 36.6, 31.5, 28.3, 27.4, 26.3, 25.8, 23.4, 22.9, 17.8, 14.3; **HRMS** (ESI+): *m*/*z* $[M+H]^+$ calcd for $C_{19}H_{32}NO_2^+$: 306.2428; found: 306.2480.

Halichonic acid B lactone (9):

[α]$^{20}_{D}$ +5.33° (*c* 0.33, $CHCl_3$); **IR** (thin film): 2963, 2930, 1777, 1454, 1375, 1325, 1275, 1219, 1195, 1168, 1146, 1101, 1063, 997, 977, 927, 906, 895, 849, 798 cm^{-1}; **^{1}H NMR** ($CDCl_3$, 400 MHz): δ = 5.41 (1H, m), 3.33 (1H, d, *J* = 12.9 Hz), 2.04 (1H, m), 1.99 (2H, m), 1.95–1.88 (4H, m), 1.71 (1H, m), 1.65 (4H, m), 1.52 (1H, dddd, *J* = 12.9 Hz, 12.9 Hz, 12.9 Hz, 3.5 Hz), 1.43 (3H, s), 1.32 (3H, s), 1.29 (1H, m), 1.25 (1H, m), 0.96 (3H, s); **^{13}C NMR** ($CDCl_3$, 100 MHz): δ = 174.8, 134.4, 120.6, 85.6, 55.6, 53.9, 52.2, 33.81, 33.78, 31.6, 27.9, 25.2, 24.6, 24.1, 23.4, 21.6, 21.2; **HRMS** (ESI+): *m*/*z* $[M+H]^+$ calcd for $C_{17}H_{28}NO_2^+$: 278.2115; found: 278.2166.

Isohalichonic acid B lactone (10):

[α]$^{20}_{D}$ –52.8° (*c* 0.37, $CHCl_3$); **IR** (solid ATR): 2965, 2928, 1778, 1460, 1372, 1328, 1275, 1210, 1161, 1142, 1091, 1066, 1021, 991, 935, 910, 888, 850, 799 cm^{-1}; **^{1}H NMR** ($CDCl_3$, 400 MHz): δ = 5.36 (1H, m), 3.51 (1H, d, *J* = 12.9 Hz), 2.08 (1H, m), 2.02–1.97 (3H, m), 1.88 (1H, m), 1.81 (1H, ddd, *J* = 12.9 Hz, 12.9 Hz, 3.2 Hz), 1.73 (1H, m), 1.66 (1H, m), 1.63 (3H, m), 1.50 (1H, m), 1.43 (3H, s), 1.42 (1H, m), 1.35 (1H, m), 1.34 (3H, s), 1.30–1.19 (1H, m), 1.06 (3H, s); **^{13}C NMR** ($CDCl_3$, 100 MHz): δ = 175.4, 134.1, 120.6, 85.4, 55.9, 54.8, 52.4, 46.3, 33.3, 31.3, 27.9, 25.9, 23.8, 23.4, 21.62, 21.60, 19.0; **HRMS** (ESI+): *m*/*z* $[M+H]^+$ calcd for $C_{17}H_{28}NO_2^+$: 278.2115; found: 278.2163.

Synthesis of (−)-halichonic acid [(−)-1]:

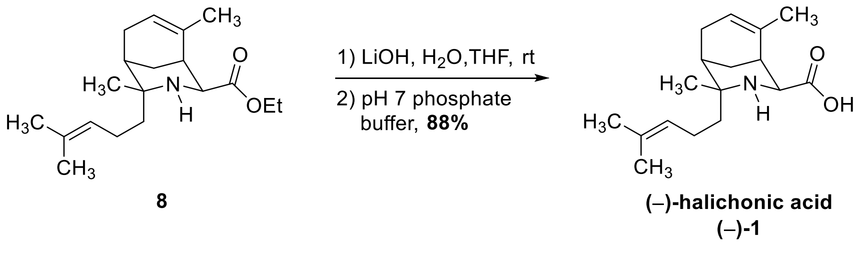

To a 50 mL flask under argon equipped with a magnetic stirring bar was added a solution of ethyl ester **8** (380.1 mg, 1.24 mmol, 1.00 equiv) in 18 mL of THF and 9 mL of water. Solid lithium hydroxide monohydrate (78.3 mg, 1.87 mmol, 1.50 equiv) was added, and the initially clear, colorless solution became cloudy. The reaction mixture was stirred at room temperature for 72 hours, at which point a clear, homogeneous solution was obtained. The reaction was quenched with 25 mL of pH 7 aqueous phosphate buffer and diluted with CH_2Cl_2. The layers were separated, and the aqueous phase was extracted with 3 × 25 mL of CH_2Cl_2, at which point the combined organic layers were dried over anhydrous Na_2SO_4. TLC (9:1 CH_2Cl_2/CH_3OH, anisaldehyde) of the dried organic phase showed complete consumption of ethyl ester **8** (R_f = 0.85) and clean formation of (−)-halichonic acid (R_f = 0.35). The drying agent was removed by vacuum filtration, and the solvent was removed under reduced pressure to give the crude product as a white solid. This material was purified by column chromatography (9:1 CH_2Cl_2/CH_3OH, anisaldehyde) to afford (−)-halichonic acid (303.8 mg, 88%) as a white amorphous powder.

mp 88–90 °C (dec.); **$[\alpha]^{20}_D$** –73.4° (*c* 0.40, CH_3OH) [lit. $[\alpha]^{21}_D$ +30° (*c* 0.40, CH_3OH) for **1**][4]; **IR** (solid ATR): 3380, 2916, 1622, 1448, 1374, 1222, 1198, 1151, 1055, 936, 903 cm^{-1}; **^{1}H NMR** (DMSO-d_6, 400 MHz): δ = 5.48 (1H, m), 5.07 (1H, m), 3.59 (1H, d, *J* = 2.7 Hz), 2.54 (1H, m), 2.21-2.11 (2H, m), 2.04 (1H, m), 1.92 (1H, m), 1.90-1.83 (2H, m), 1.75 (1H, m), 1.63 (3H, s), 1.59 (3H, s), 1.55 (3H, s), 1.50 (1H, m), 1.48 (1H, m), 1.26 (3H, s); **^{13}C NMR** (DMSO-d_6, 100 MHz): δ = 170.3, 132.2, 131.1, 124.7, 124.0, 58.8, 56.1, 34.8, 34.4, 29.8, 26.7, 26.2, 25.5, 23.6, 23.3, 22.1, 17.5; **HRMS** (ESI+): *m*/*z* $[M+H]^+$ calcd for $C_{17}H_{28}NO_2^+$: 278.2115; found: 278.2169.

Synthesis of (−)-halichonic acid B [(−)-2]:

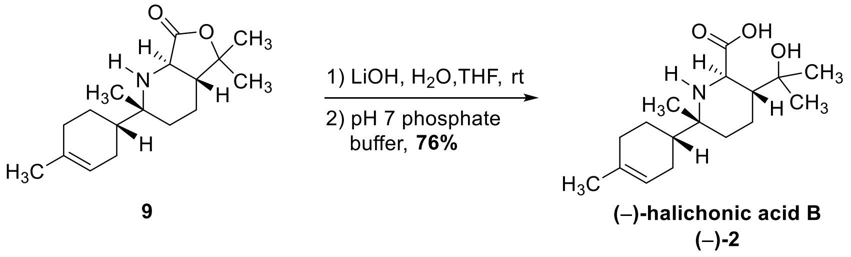

To a 25 mL flask under argon equipped with a magnetic stirring bar was added a solution of lactone **9** (57.2 mg, 0.206 mmol, 1.00 equiv) in 3 mL of THF and 1.5 mL of water. Solid lithium hydroxide monohydrate (10.8 mg, 0.258 mmol, 1.25 equiv) was added, and the resulting colorless, homogeneous reaction mixture was stirred at room temperature for 24 hours. After this time, the reaction was quenched with 10 mL of pH 7 aqueous phosphate buffer and diluted with CH_2Cl_2. The layers were separated, and the aqueous phase was repeatedly extracted with 8 × 10 mL of CH_2Cl_2, at which point the combined organic layers were dried over anhydrous Na_2SO_4. [Note: The product is water-soluble and partitions between the aqueous and organic phases. In order to achieve the maximum recovery of material, extraction of the aqueous phase with CH_2Cl_2 should be continued until TLC shows no additional product in the organic extracts.). TLC (9:1 CH_2Cl_2/CH_3OH, anisaldehyde) of the dried organic phase showed complete consumption of lactone **9** (R_f = 0.69) and clean formation of (−)-halichonic acid B (R_f = 0.27). The drying agent was removed by vacuum filtration, and the solvent was removed under reduced pressure to give the crude product as a white solid. This material was purified by column chromatography (9:1 CH_2Cl_2/CH_3OH, anisaldehyde) to afford (−)-halichonic acid B (46.3 mg, 76%) as a white amorphous powder.

mp 172–173 °C (dec.); **$[\alpha]^{20}_D$** –21.5° (*c* 0.21, CH_3OH) [lit. $[\alpha]^{21}_D$ +47° (*c* 0.40, CH_3OH) for **2**][5]; IR (solid ATR): 3385, 2967, 2931, 1632, 1439, 1386, 1177 cm^{-1}; **^{1}H NMR** (CD_3OD, 400 MHz): δ = 5.45 (1H, m), 3.58 (1H, d, J = 10.3 Hz), 2.16 (1H, m), 2.12 (1H, m), 2.04–2.02 (4H, m),1.93 (1H, m), 1.86 (1H, m), 1.75 (1H, m), 1.67 (3H, m), 1.62 (1H, m), 1.57 (1H, m), 1.30 (1H, m), 1.31 (3H, s), 1.27 (3H, s), 1.24 (3H, s); **^{13}C NMR** (CD_3OD, 100 MHz): δ = 174.2, 135.4, 120.1, 72.6, 61.0, 58.8, 47.8, 36.3, 32.1, 31.6, 28.9, 25.9, 25.5, 24.8, 23.4, 22.2, 20.7; **HRMS** (ESI+): m/z $[M+H]^+$ calcd for $C_{17}H_{30}NO_3^+$: 296.2220; found: 296.2274.

Synthesis of (−)-isohalichonic acid B (11):

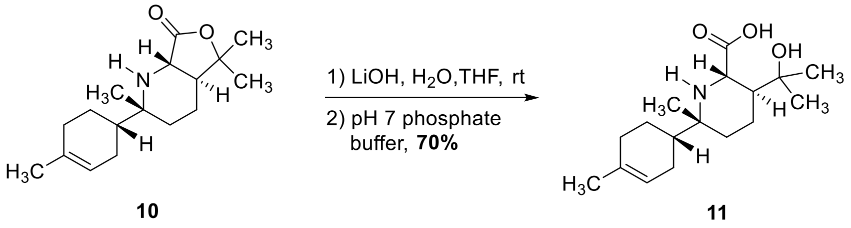

To a 25 mL flask under argon equipped with a magnetic stirring bar was added a solution of lactone **10** (78.7 mg, 0.284 mmol, 1.00 equiv) in 4 mL of THF and 2 mL of water. Solid lithium hydroxide monohydrate (14.9 mg, 0.355 mmol, 1.25 equiv) was added, and the resulting colorless, homogeneous reaction mixture was stirred at room temperature for 24 hours. After this time, the reaction was quenched with 10 mL of pH 7 aqueous phosphate buffer and diluted with CH_2Cl_2. The layers were separated, and the aqueous phase was repeatedly extracted with 8 × 10 mL of CH_2Cl_2, at which point the combined organic layers were dried over anhydrous Na_2SO_4. [Note: The product is water-soluble and partitions between the aqueous and organic phases. In order to achieve the maximum recovery of material, extraction of the aqueous phase with CH_2Cl_2 should be continued until TLC shows no additional product in the organic extracts.). TLC (9:1 CH_2Cl_2/CH_3OH, anisaldehyde) of the dried organic phase showed complete consumption of lactone **10** (R_f = 0.88) and clean formation of **11** (R_f = 0.21). The drying agent was removed by vacuum filtration, and the solvent was removed under reduced pressure to give the crude product as a white solid. This material was purified by column chromatography (9:1 CH_2Cl_2/CH_3OH, anisaldehyde) to afford **11** (58.7 mg, 70%) as a white amorphous powder.

mp 183–184 °C (dec.); **$[\alpha]^{20}_D$** –49.6° (*c* 0.28, CH_3OH); **IR** (solid ATR): 3388, 2965, 2926, 1629, 1435, 1382, 1173, 918, 717 cm^{-1}; **^1H NMR** (CD_3OD, 400 MHz): δ = 5.42 (1H, m), 3.63 (1H, d, *J* = 11.3 Hz), 2.04 (4H, m), 2.03 (1H, m), 1.94 (1H, m), 1.89 (1H, m), 1.87 (1H, m), 1.84 (1H, m), 1.67 (3H, m), 1.62–1.47 (2H, m), 1.38 (3H, s), 1.30 (1H, m), 1.24 (3H, s), 1.22 (3H, s); **^{13}C NMR** (CD_3OD, 100 MHz): δ = 175.0, 135.3, 120.1, 72.7, 60.8, 59.1, 49.5, 44.9, 33.7, 31.6, 29.2, 26.2, 25.1, 24.6, 23.5, 23.4, 14.5; **HRMS** (ESI+): *m*/*z* $[M+H]^+$ calcd for $C_{17}H_{30}NO_3^+$: 296.2220; found: 296.2273.

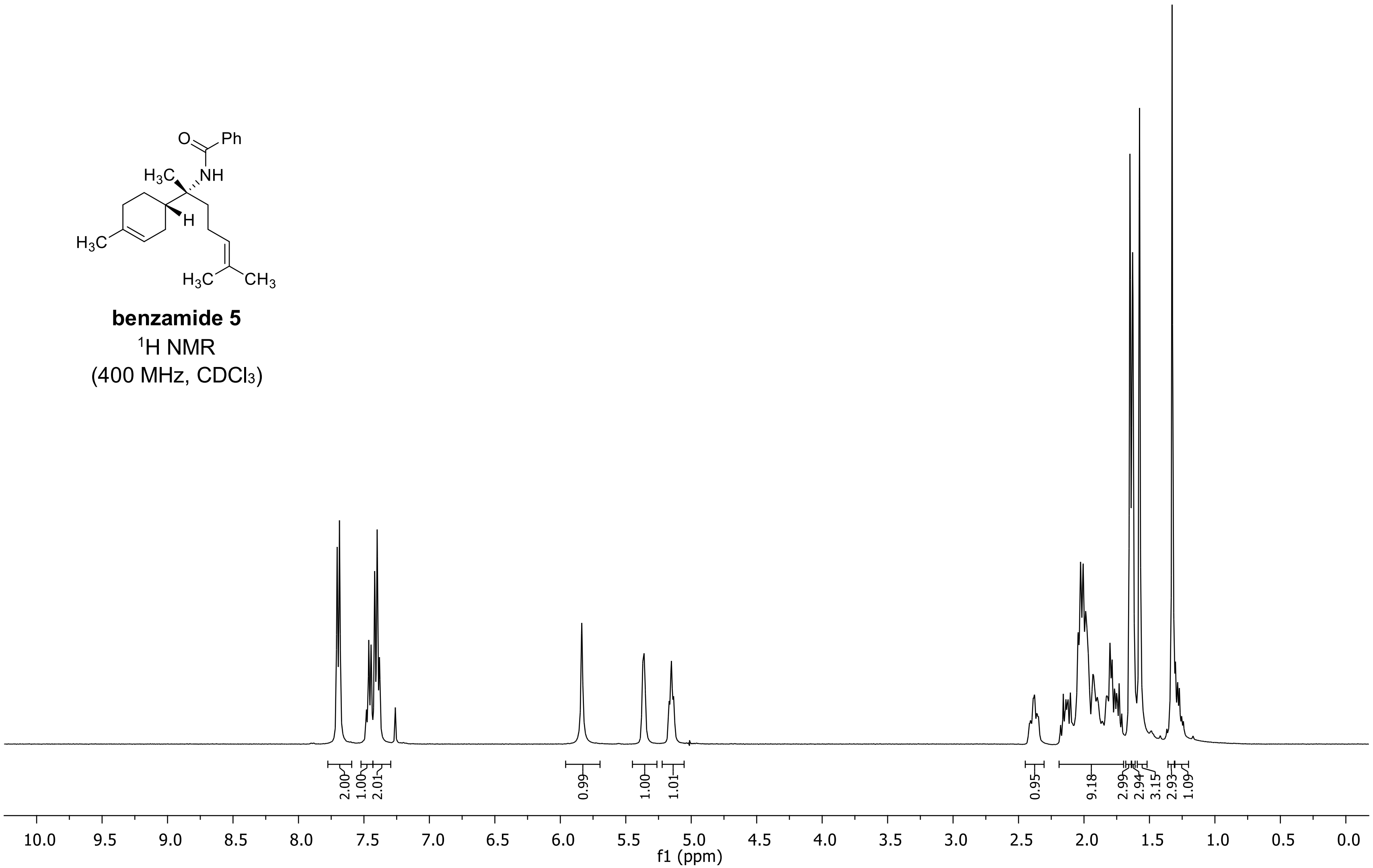

benzamide 5

^{1}H NMR

(400 MHz, $CDCl_3$)

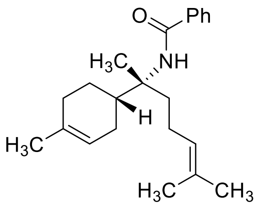

benzamide 5

^{13}C NMR

(100 MHz, $CDCl_3$)

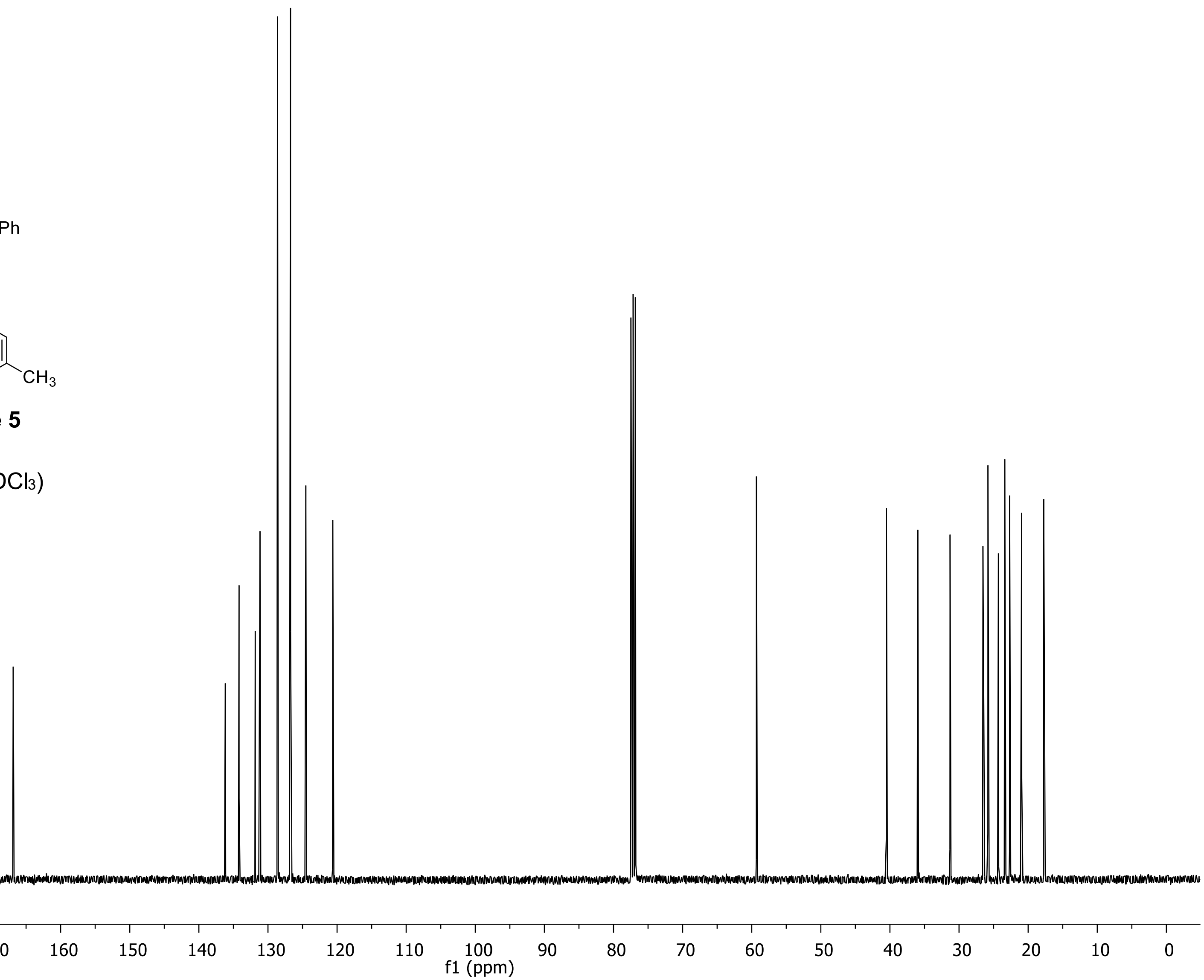

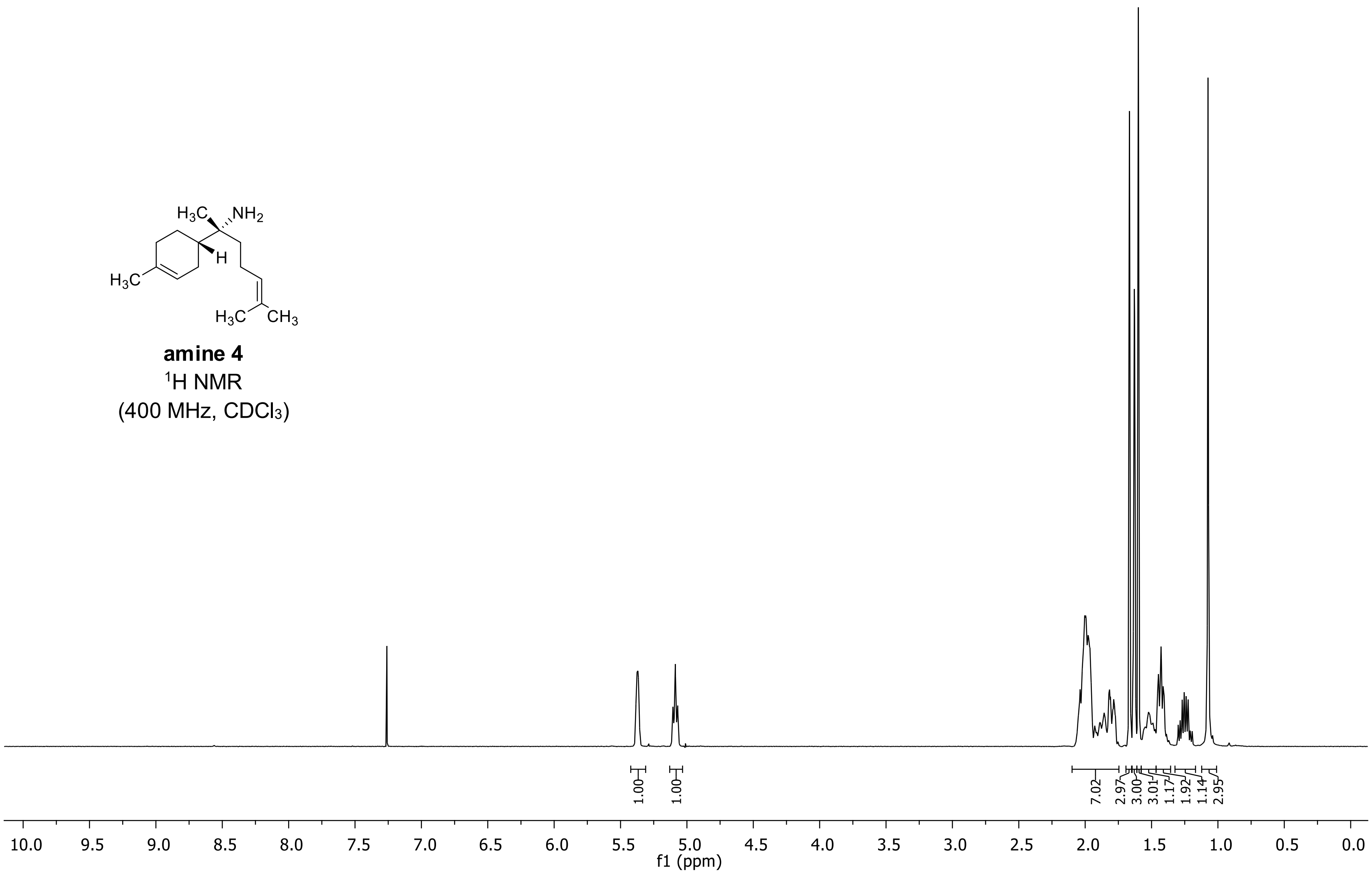

amine 4

^{1}H NMR

(400 MHz, $CDCl_3$)

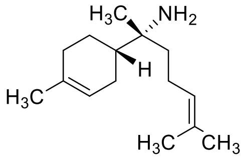

amine 4

^{13}C NMR

(100 MHz, $CDCl_3$)

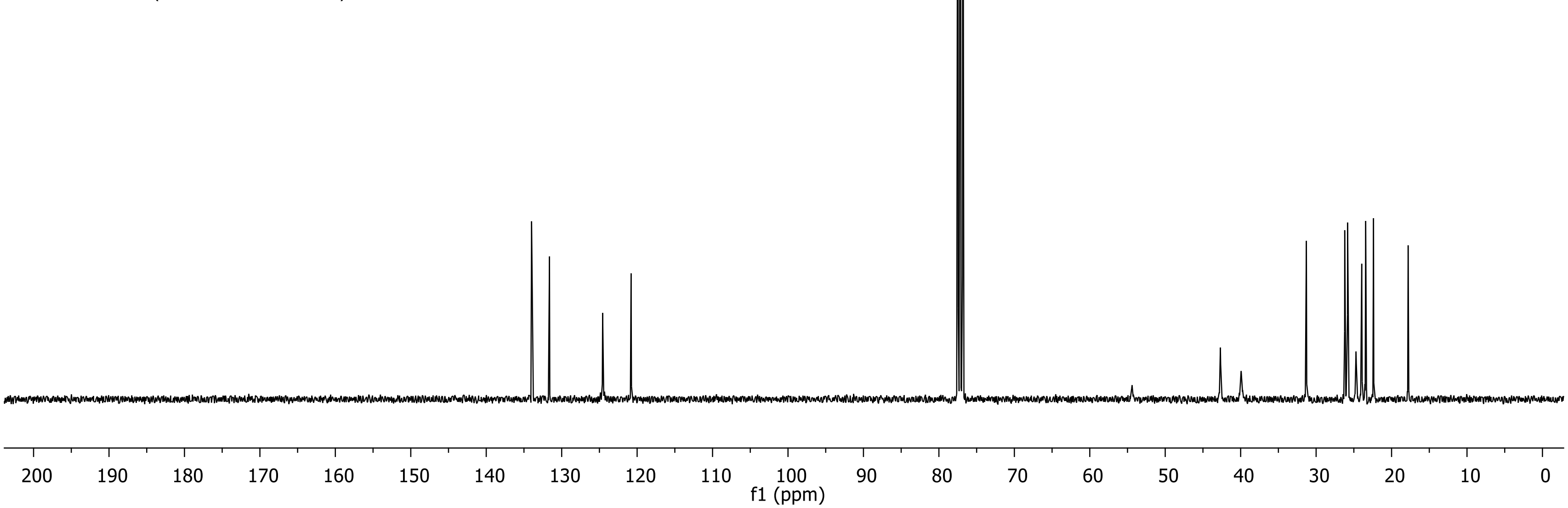

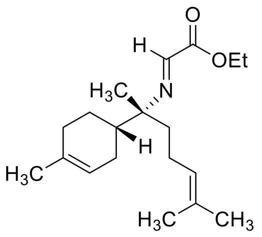

imine 7

^1H NMR

(400 MHz, $CDCl_3$)

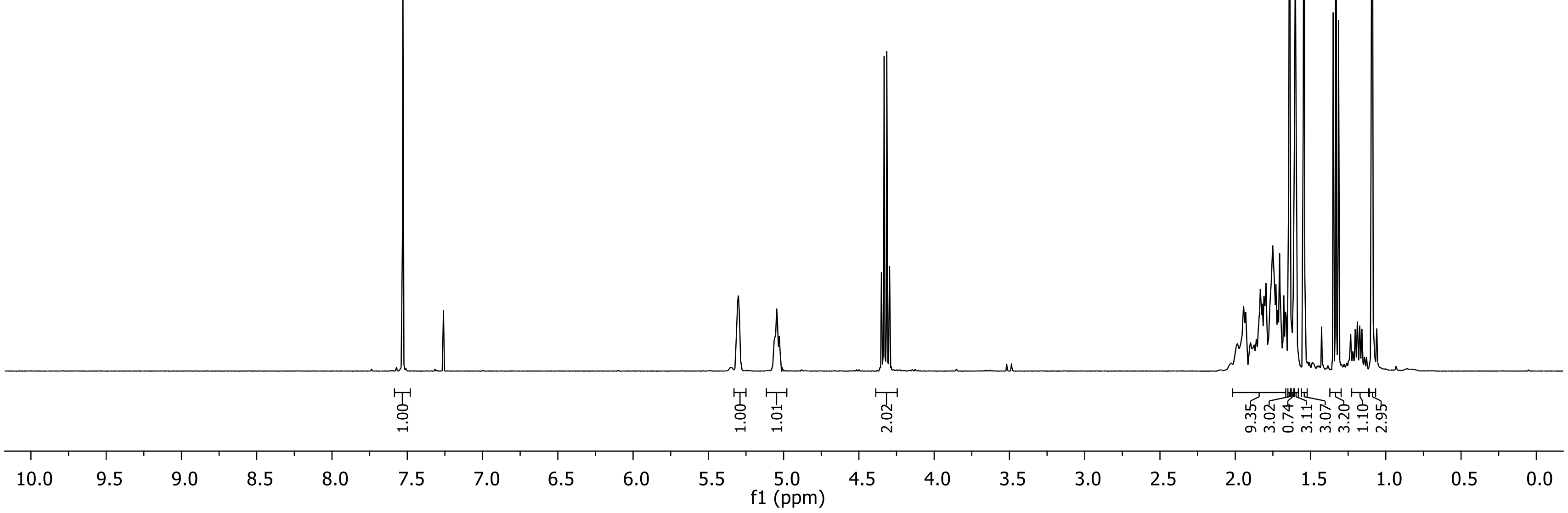

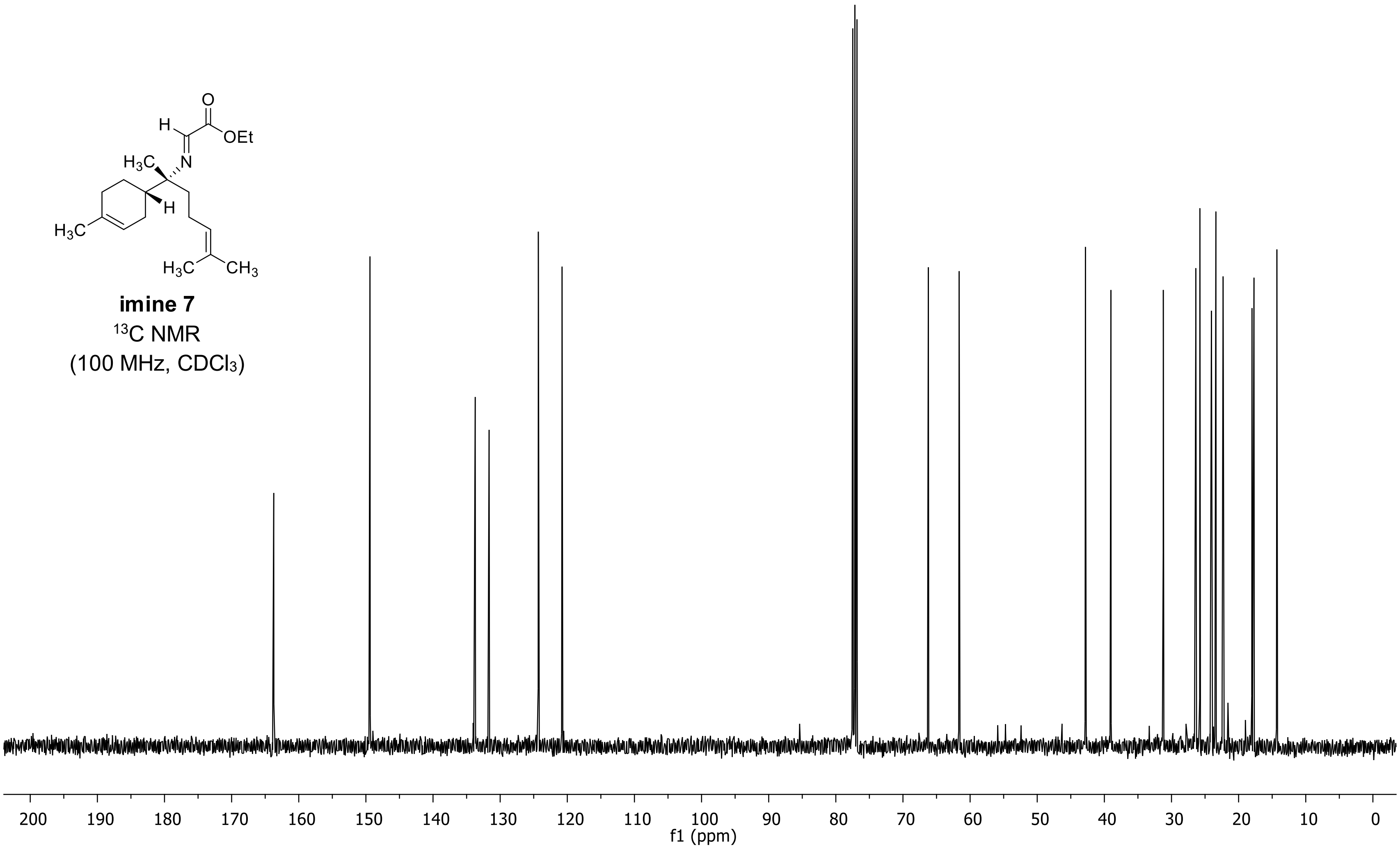
O
H
OEt
H_3C
N
H
H_3C
H_3C
CH_3
imine 7
^{13}C NMR
(100 MHz, $CDCl_3$)
200
190
180
170
160
150
140
130
120
110
100
90
80
70
60
50
40
30
20
10
0
f1 (ppm)

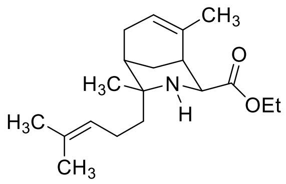

ethyl ester 8

^{1}H NMR

(400 MHz, $CDCl_3$)

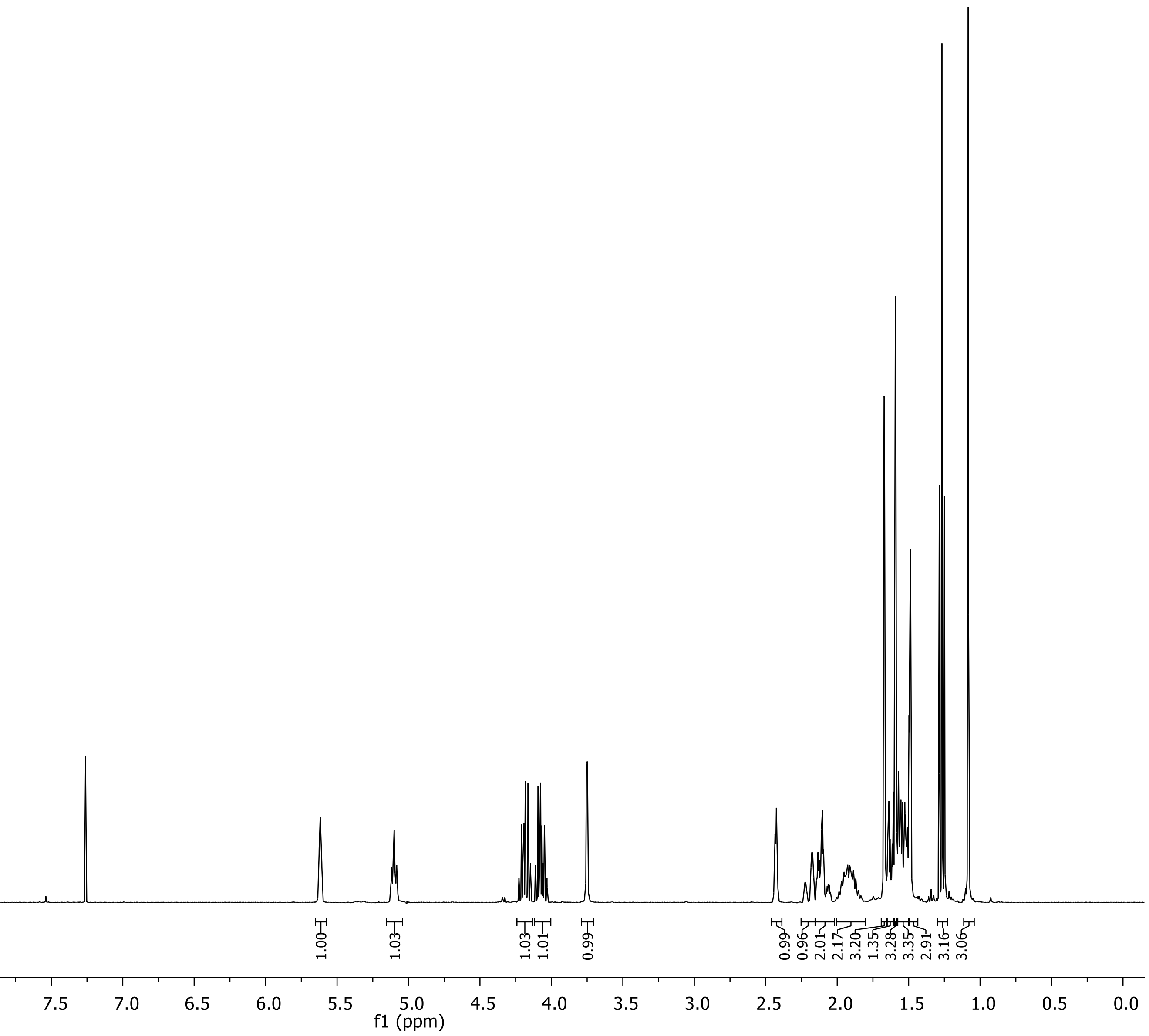

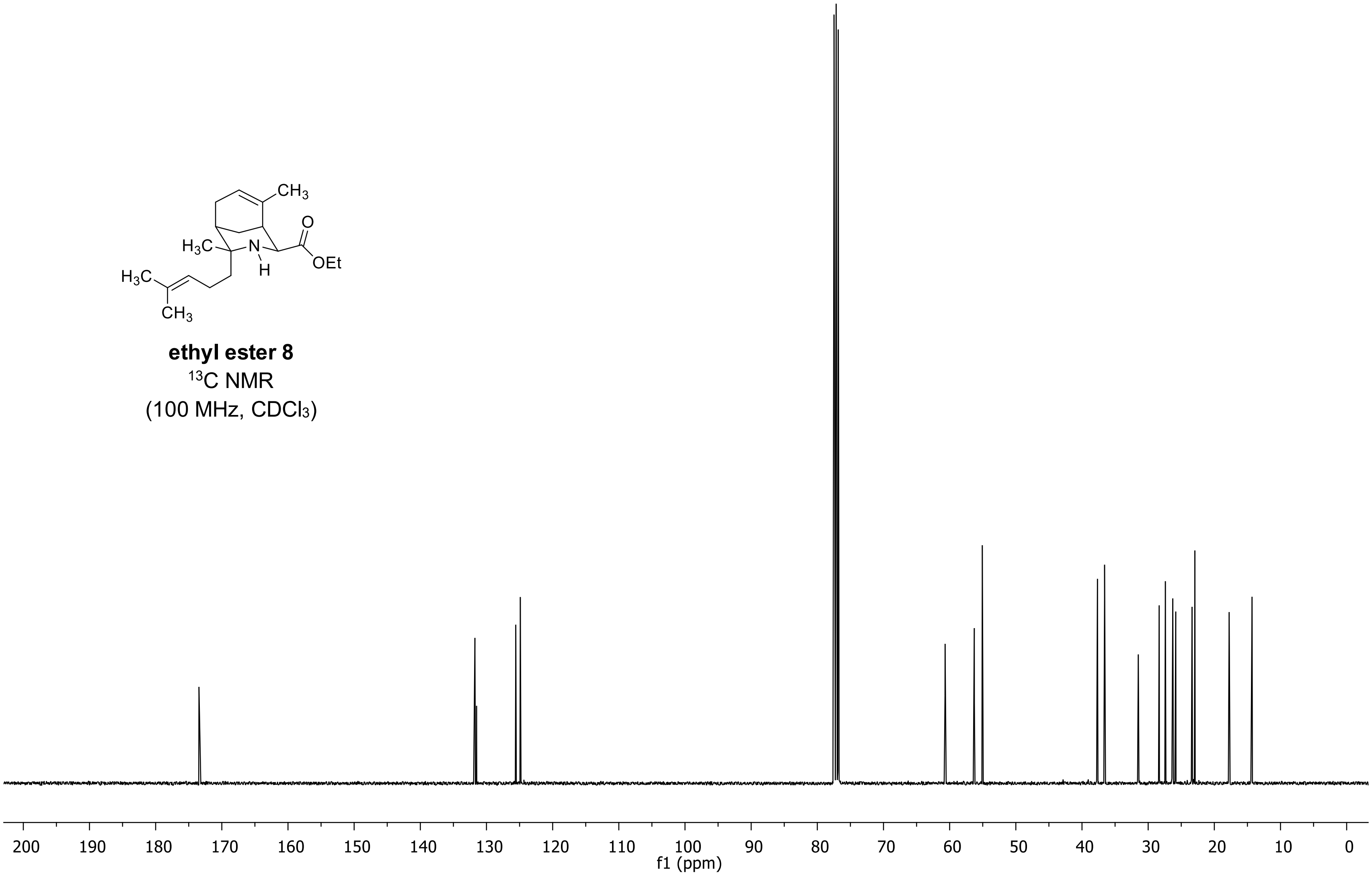

ethyl ester 8

^{13}C NMR

(100 MHz, $CDCl_3$)

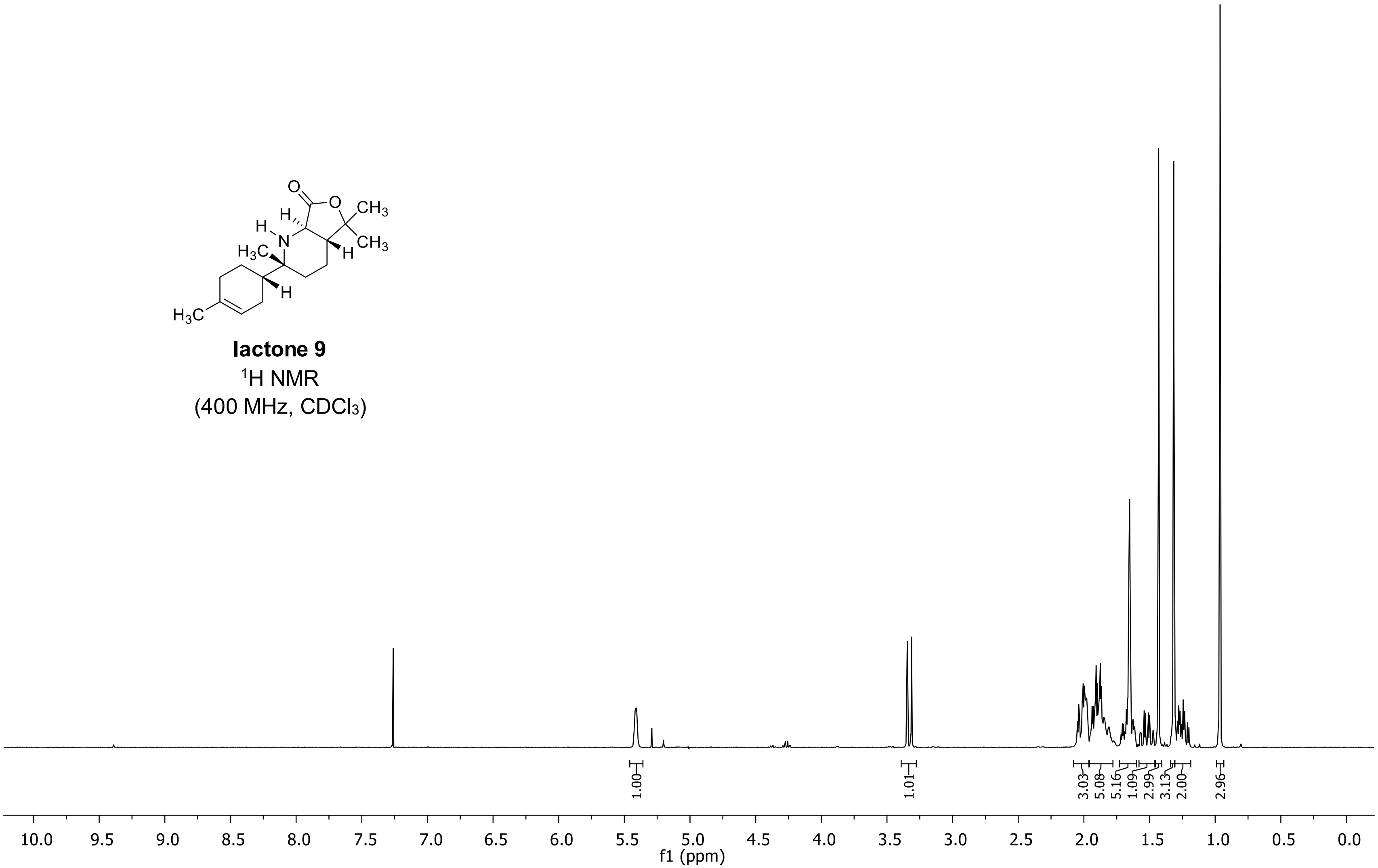

lactone 9

^{1}H NMR

(400 MHz, $CDCl_3$)

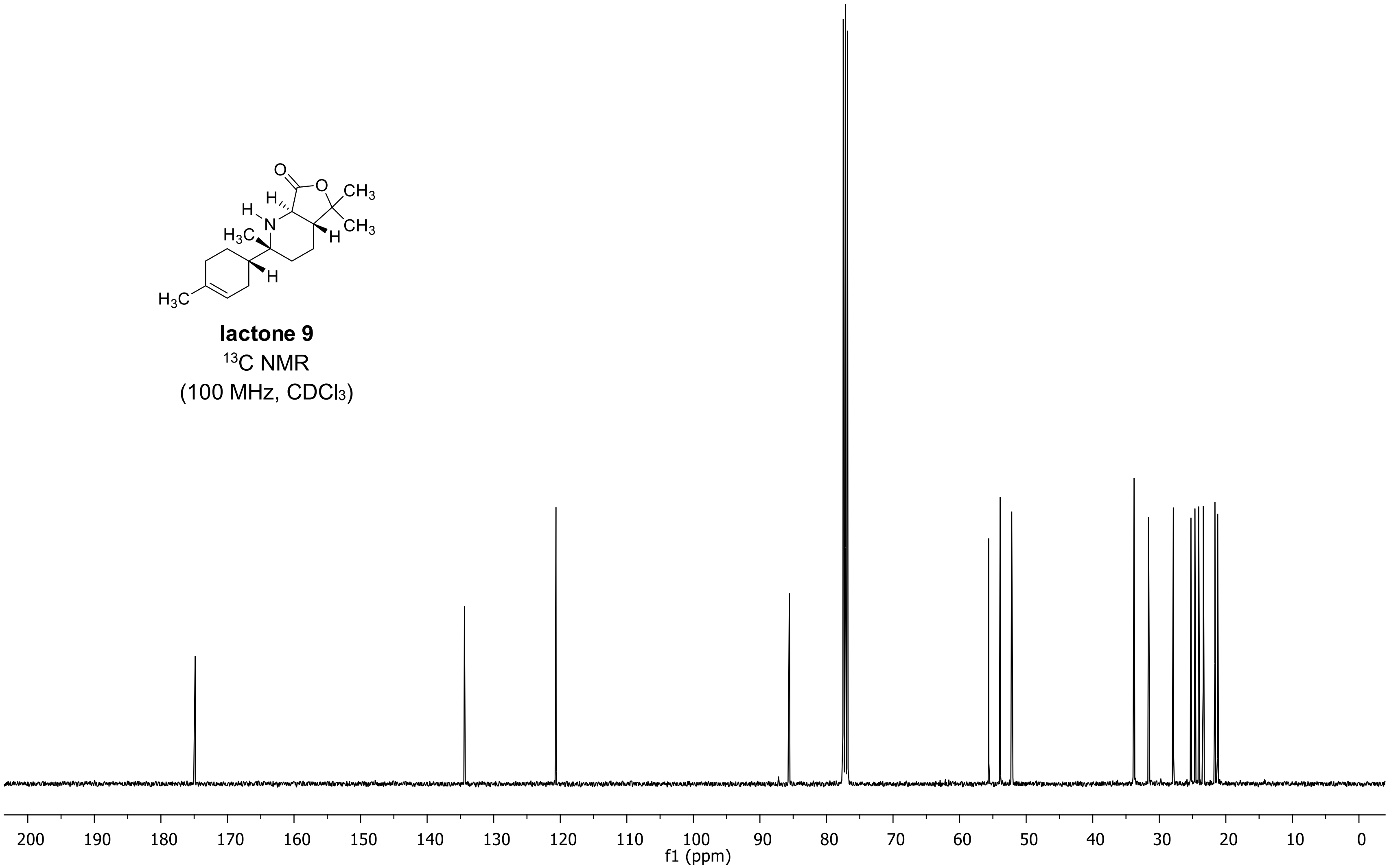
O
O
CH3
CH3
H
H
N
H3C
H
H
H3C
lactone 9
13C NMR
(100 MHz, CDCl3)
200
190
180
170
160
150
140
130
120
110
100
90
80
70
60
50
40
30
20
10
0
f1 (ppm)

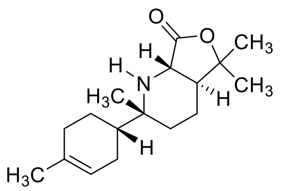

lactone 10

^{1}H NMR

(400 MHz, $CDCl_3$)

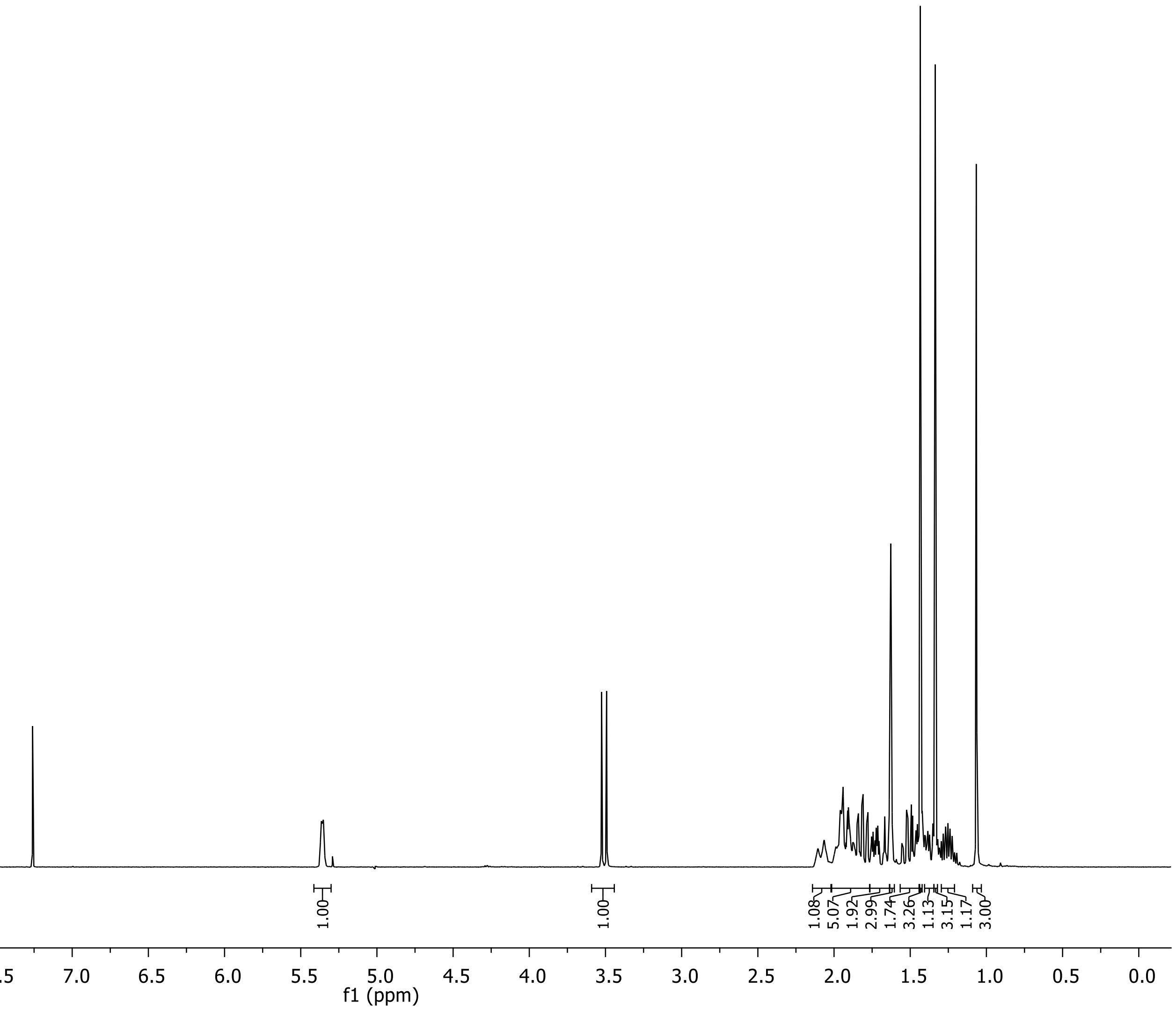

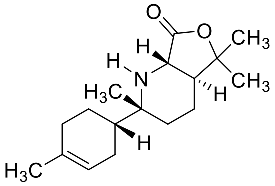

lactone 10

^{13}C NMR

(100 MHz, $CDCl_3$)

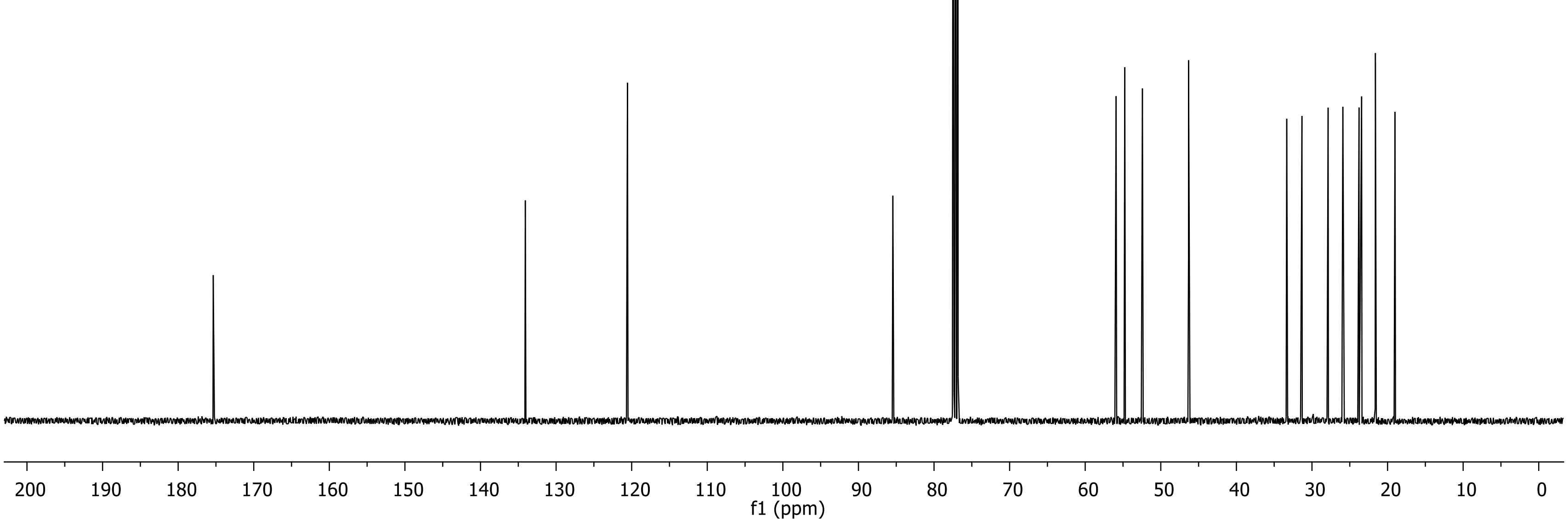

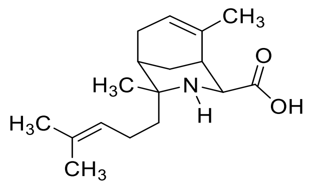

(−)-halichonic acid [(−)-1]

^{1}H NMR

(400 MHz, DMSO-d_6)

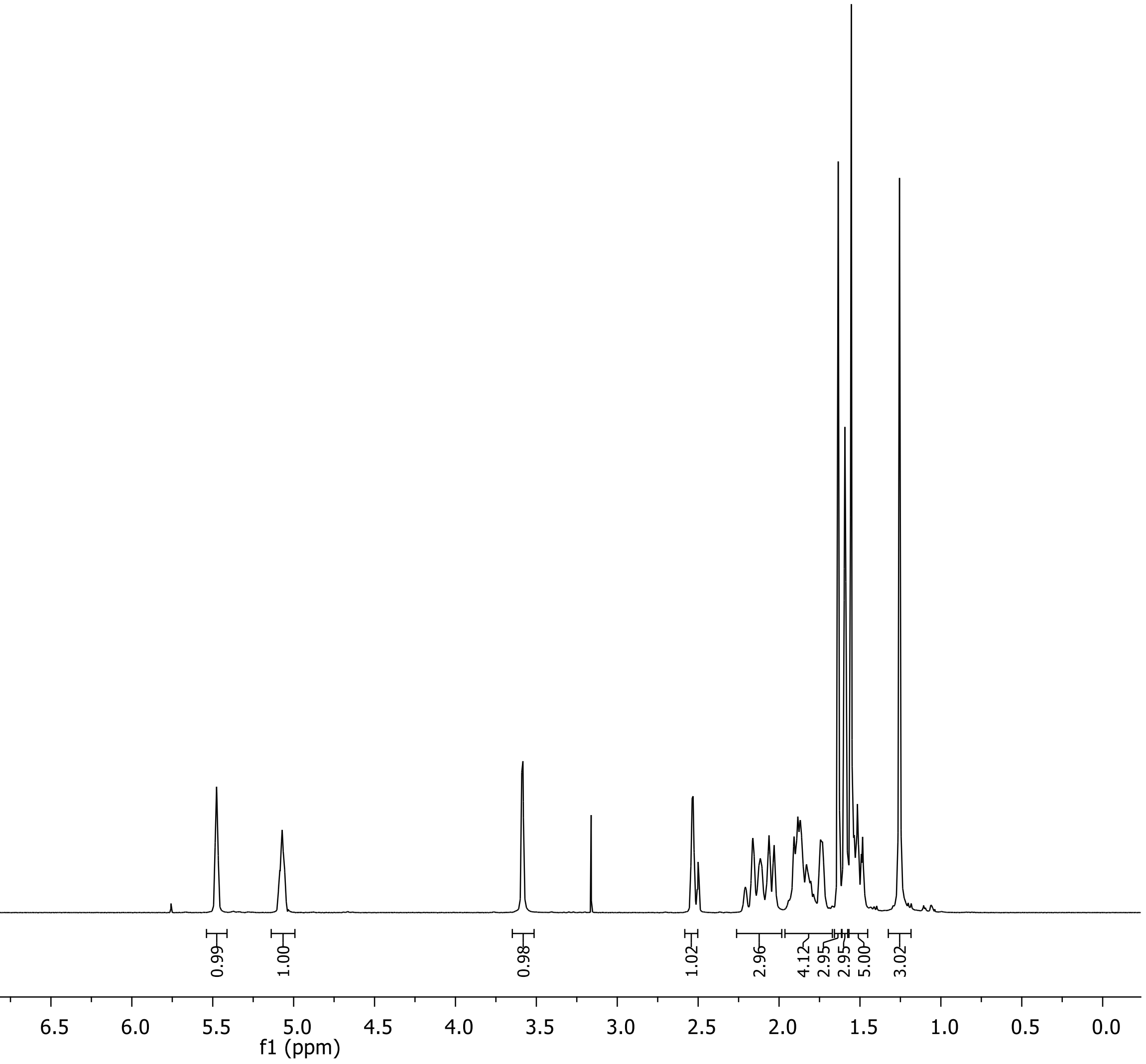

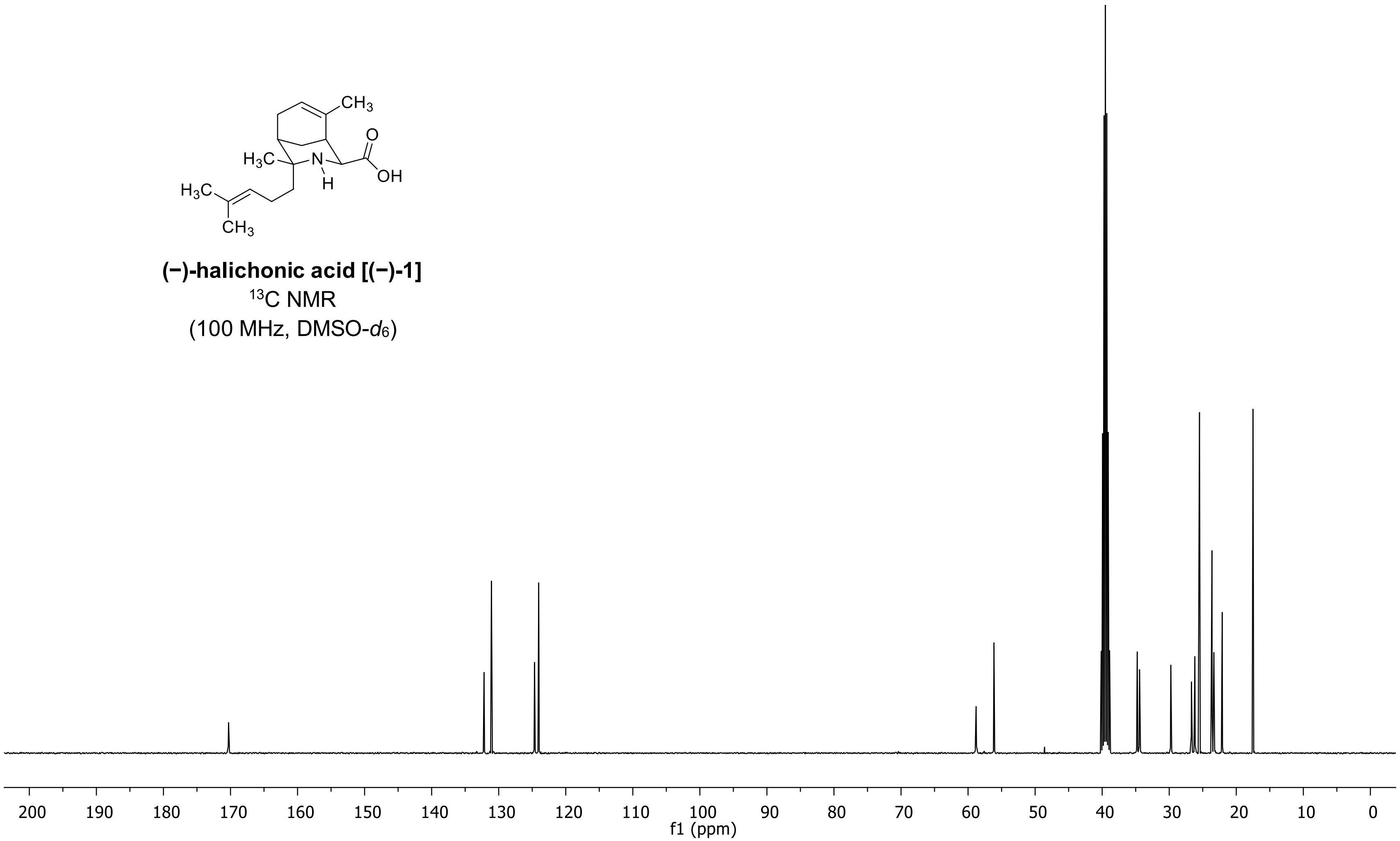

(−)-halichonic acid [(−)-1]

^{13}C NMR

(100 MHz, DMSO-d_6)

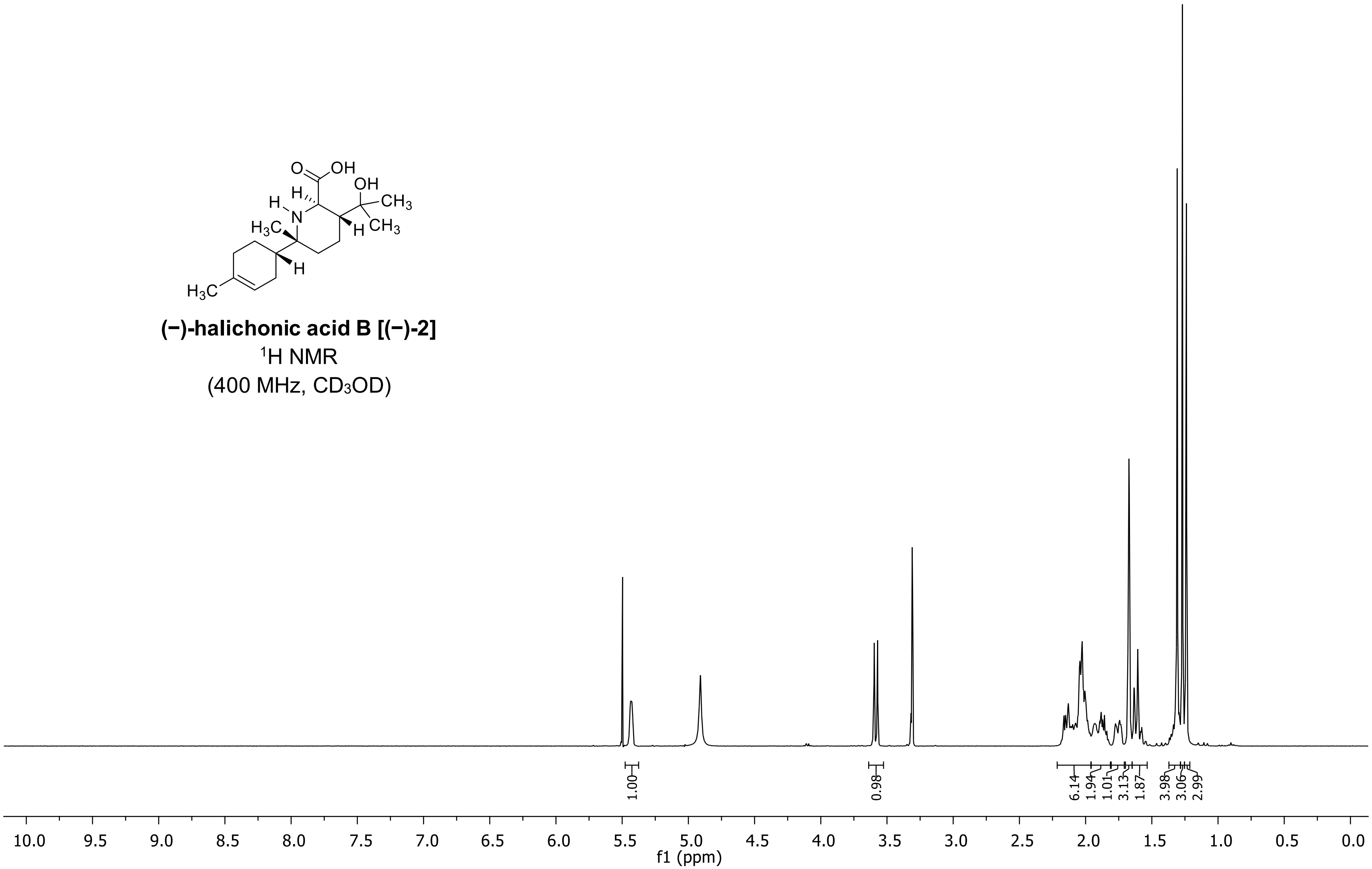

(−)-halichonic acid B [(−)-2]

^{1}H NMR

(400 MHz, CD_3OD)

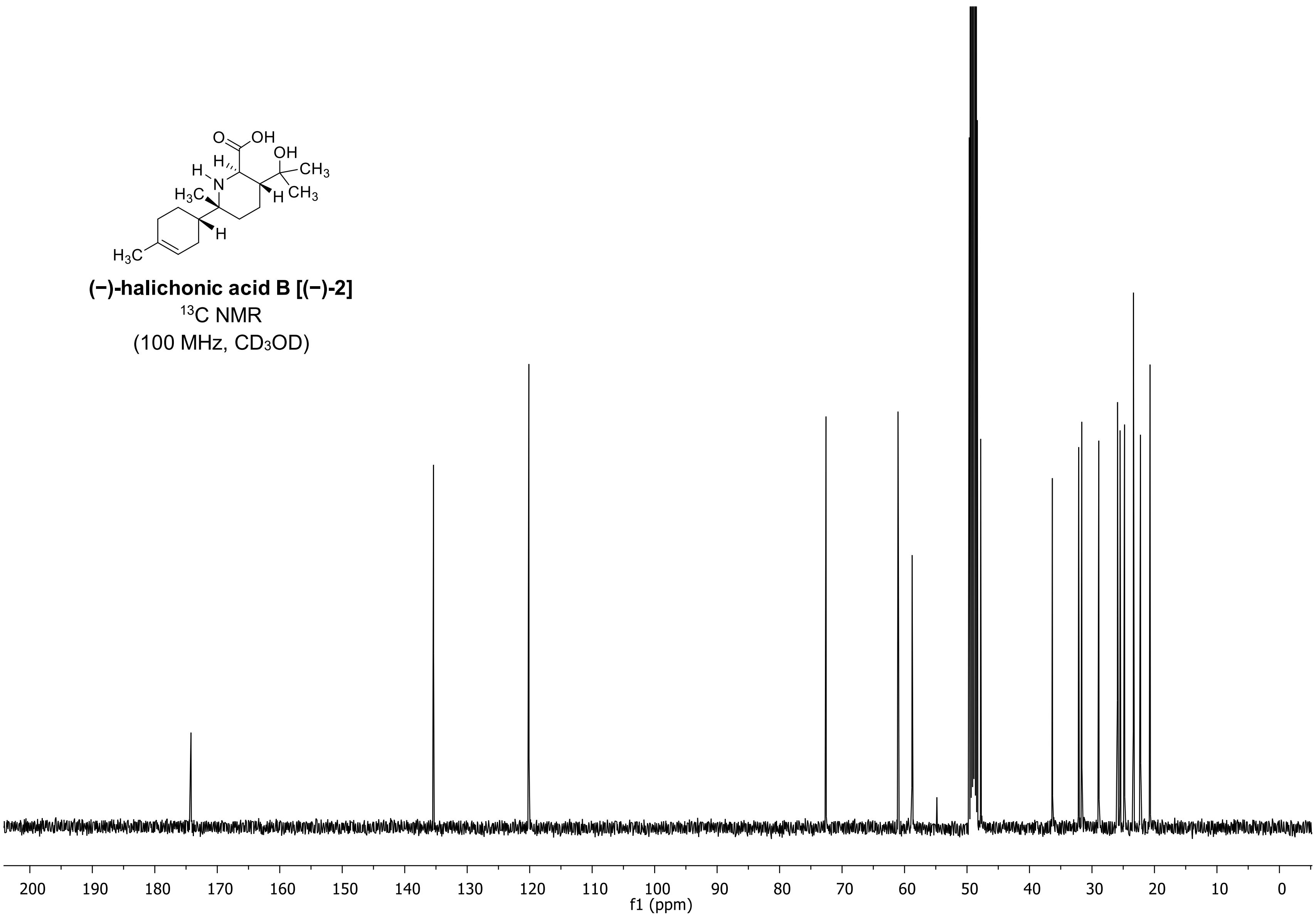

(−)-halichonic acid B [(−)-2]

^{13}C NMR

(100 MHz, CD_3OD)

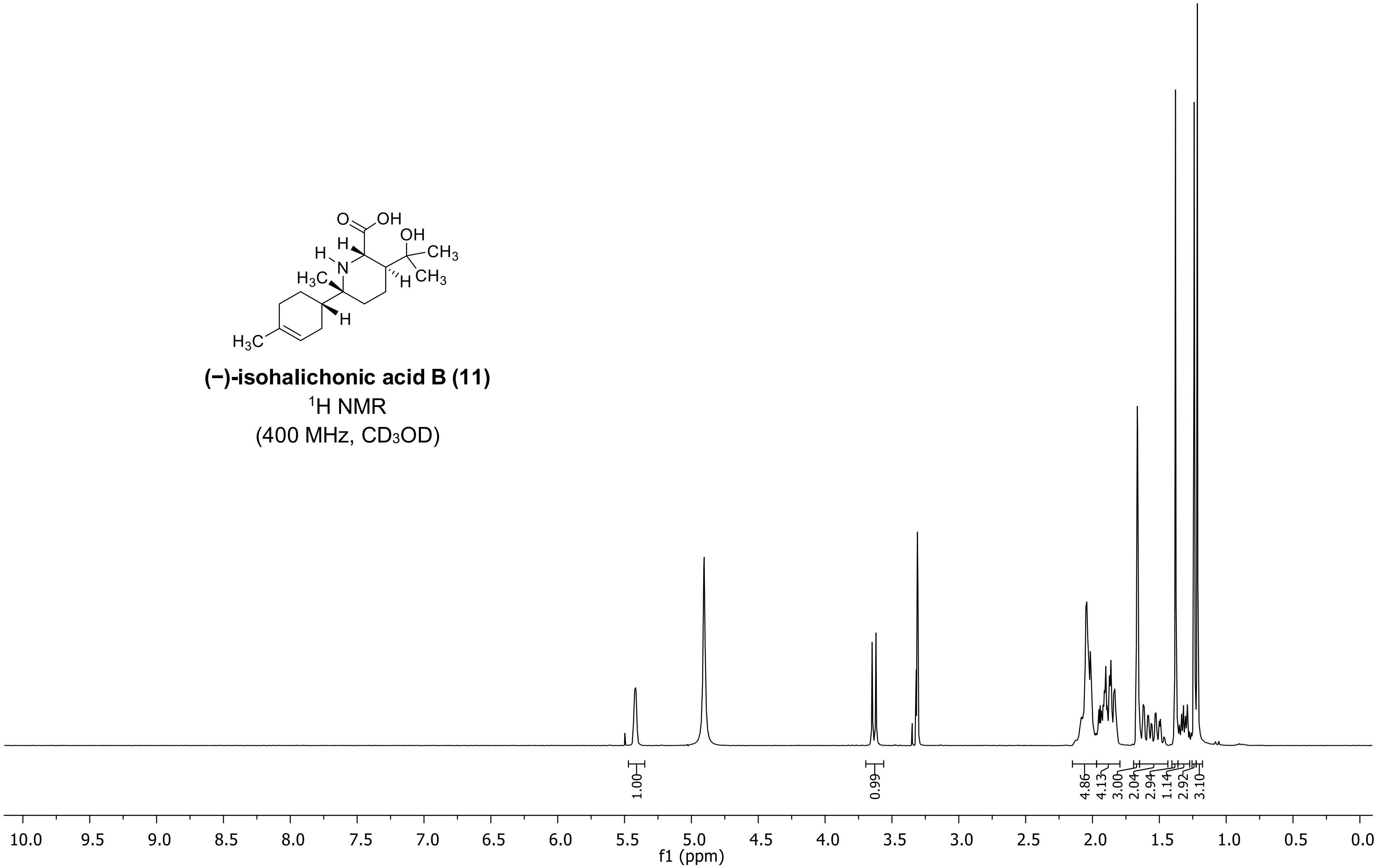

(−)-isohalichonic acid B (11)

^{1}H NMR

(400 MHz, CD_3OD)

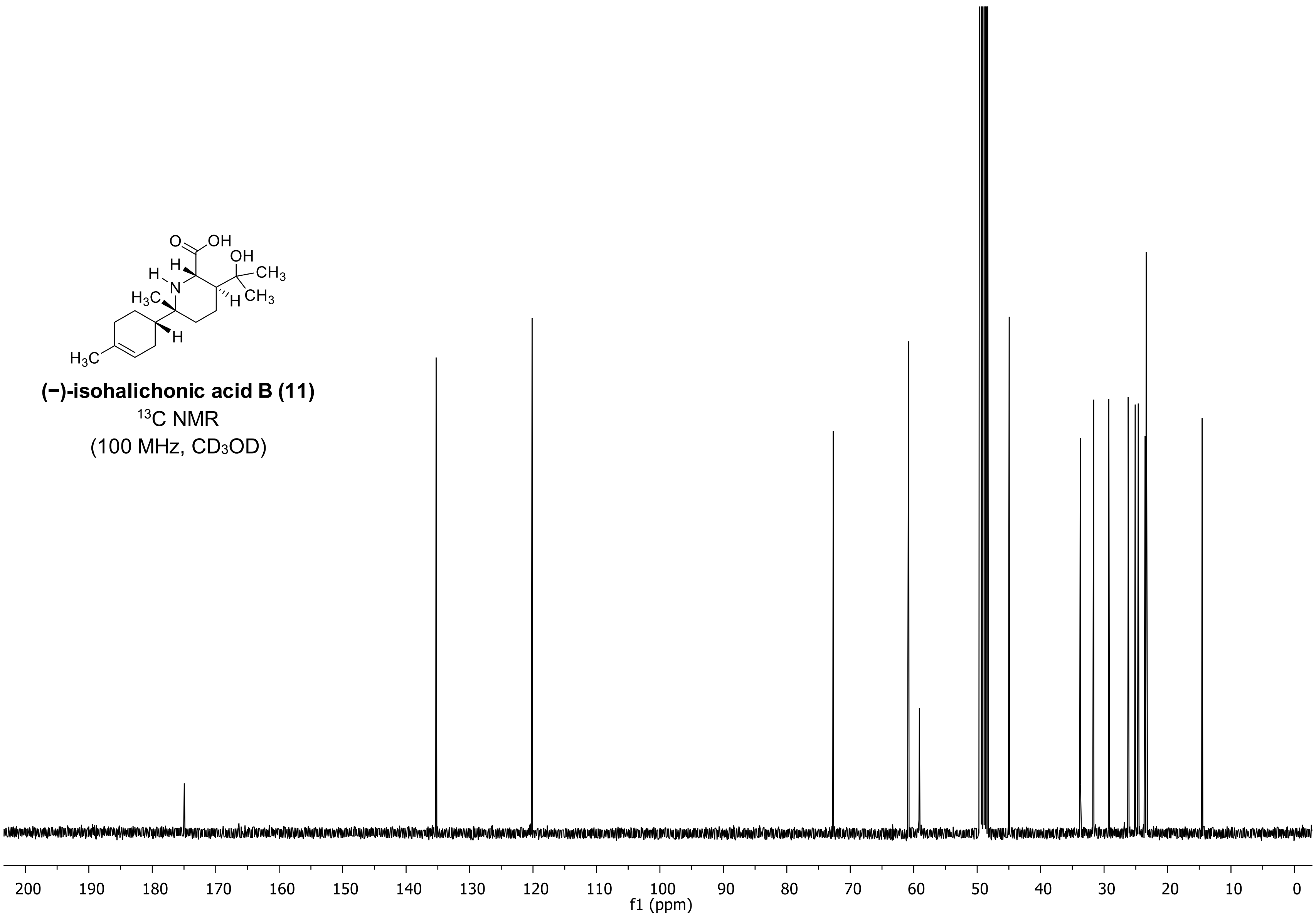

(−)-isohalichonic acid B (11)

^{13}C NMR

(100 MHz, CD_3OD)

Complete ^{1}H and ^{13}C NMR assignments for (−)-halichonic acid ethyl ester (**8**)

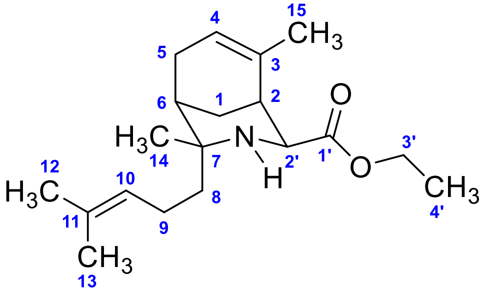

(–)-halichonic acid ethyl ester

	^{13}C NMR (100 MHz, $CDCl_3$)	^{1}H NMR (400 MHz, $CDCl_3$)
**C1**	28.3	1.62 (1H, m)<br>2.11 (1H, m)
**C2**	37.7	2.43 (1H, m)
**C3**	131.8	N/A
**C4**	125.6	5.62 (1H, m)
**C5**	27.4	2.11 (1H, m)<br>2.20 (1H, m)
**C6**	31.5	1.50 (1H, m)
**C7**	55.1	N/A
**C8**	36.6	1.57–1.54 (2H, m)
**C9**	22.9	2.00–1.84 (2H, m)
**C10**	124.9	5.10 (1H, m)
**C11**	131.5	N/A
**C12**	25.8	1.67 (3H, d, J = 0.9 Hz)
**C13**	17.8	1.59 (3H, s)
**C14**	26.3	1.08 (3H, s)
**C15**	23.4	1.49 (3H, m)
**C1'**	173.5	N/A
**C2'**	56.3	3.75 (1H, d, J = 2.8 Hz)
**C3'**	60.7	4.07 (1H, dq, J = 10.8 Hz, 7.2 Hz)<br>4.19 (1H, dq, J = 10.8 Hz, 7.2 Hz)
**C4'**	14.3	1.27 (3H, t, J = 7.2 Hz)

Complete ^{1}H and ^{13}C NMR Assignments for (−)-halichonic acid B lactone (**9**)

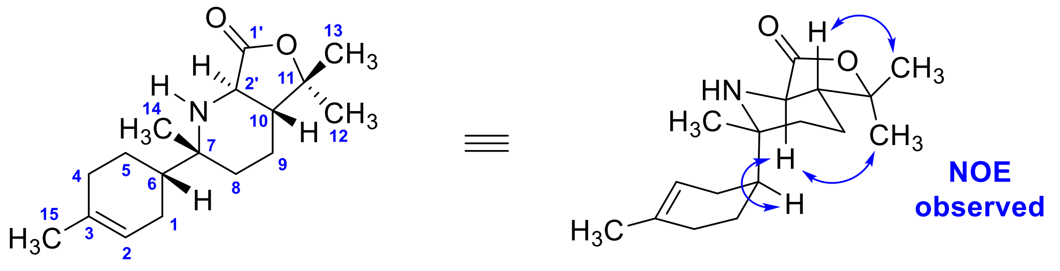

	^{13}C NMR (100 MHz, $CDCl_3$)	^{1}H NMR (400 MHz, $CDCl_3$)
**C1**	24.6	1.95–1.88 (2H, m)
**C2**	120.6	5.41 (1H, m)
**C3**	134.4	N/A
**C4**	31.6	1.99 (2H, m)
**C5**	25.2	1.29 (1H, m)<br>1.65 (1H, m)
**C6**	33.78	1.94 (1H, m)
**C7**	55.6	N/A
**C8**	33.81	1.25 (1H, m)<br>2.04 (1H, m)
**C9**	21.2	1.52 (1H, dddd, *J* = 12.9 Hz, 12.9 Hz, 12.9 Hz, 3.5 Hz)<br>1.71 (1H, m)
**C10**	52.2	1.94 (1H, m)
**C11**	85.6	N/A
**C12**	21.6	1.32 (3H, s)
**C13**	27.9	1.43 (3H, s)
**C14**	24.1	0.96 (3H, s)
**C15**	23.4	1.65 (3H, m)
**C1’**	174.8	N/A
**C2’**	53.9	3.33 (1H, d, *J* = 12.9 Hz)

Complete ^{1}H and ^{13}C NMR Assignments for isohalichonic acid B lactone (**10**)

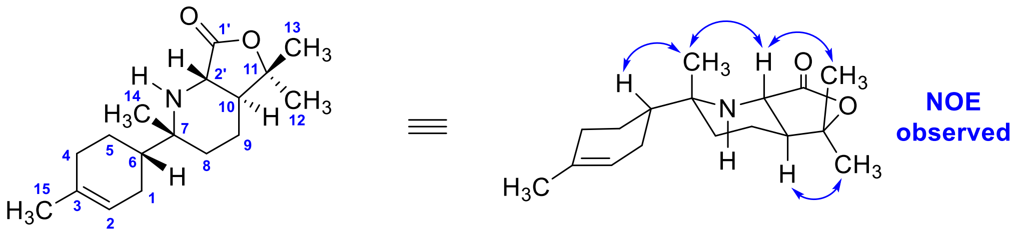

isohalichonic acid B lactone (10)

	^{13}C NMR (100 MHz, $CDCl_3$)	^{1}H NMR (400 MHz, $CDCl_3$)
**C1**	23.8	1.30–1.19 (1H, m)<br>1.94 (1H, m)
**C2**	120.6	5.36 (1H, m)
**C3**	134.1	N/A
**C4**	31.3	2.02–1.97 (2H, m)
**C5**	25.9	1.88 (1H, m)<br>2.08 (1H, m)
**C6**	46.3	1.42 (1H, m)
**C7**	55.9	N/A
**C8**	33.3	1.35 (1H, m)<br>1.66 (1H, m)
**C9**	21.60	1.50 (1H, m)<br>1.73 (1H, m)
**C10**	52.4	1.81 (1H, ddd, *J* = 12.9 Hz, 12.9 Hz, 3.2 Hz)
**C11**	85.4	N/A
**C12**	27.9	1.43 (3H, s)
**C13**	21.62	1.34 (3H, s)
**C14**	19.0	1.06 (3H, s)
**C15**	23.4	1.63 (3H, m)
**C1’**	175.4	N/A
**C2’**	54.8	3.51 (1H, d, *J* = 12.9 Hz)

Complete ^{1}H and ^{13}C NMR assignments for (−)-halichonic acid [(−)-**1**]

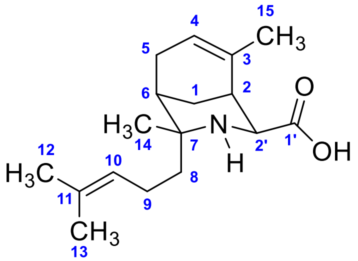

(–)-halichonic acid

	**Reported ^{13}C NMR (150 MHz, DMSO-d_6)[4]**	**Observed ^{13}C NMR (100 MHz, DMSO-d_6)**	**Reported ^{1}H NMR (600 MHz, DMSO-d_6)[4]**	**Observed ^{1}H NMR (400 MHz, DMSO-d_6)**
**C1**	26.6	26.7	1.50 (1H, m)<br>2.04 (1H, m)	1.50 (1H, m)<br>2.04 (1H, m)
**C2**	34.6	34.8	2.53 (1H, m)	2.54 (1H, m)
**C3**	132.1	132.2	N/A	N/A
**C4**	124.7	124.7	5.47 (1H, m)	5.48 (1H, m)
**C5**	26.1	26.2	2.11 (1H, m)<br>2.17 (1H, m)	2.21-2.11 (2H, m)
**C6**	29.7	29.8	1.75 (1H, m)	1.75 (1H, m)
**C7**	58.9	58.8	N/A	N/A
**C8**	34.3	34.4	1.49 (1H, m)<br>1.91 (1H, m)	1.48 (1H, m)<br>1.92 (1H, m)
**C9**	22.0	22.1	1.82 (1H, m)<br>1.90 (1H, m)	1.90-1.83 (2H, m)
**C10**	123.9	124.0	5.07 (1H, m)	5.07 (1H, m)
**C11**	131.1	131.1	N/A	N/A
**C12**	25.4	25.5	1.63 (3H, s)	1.63 (3H, s)
**C13**	17.5	17.5	1.55 (3H, s)	1.55 (3H, s)
**C14**	23.2	23.3	1.25 (3H, s)	1.26 (3H, s)
**C15**	23.6	23.6	1.59 (3H, s)	1.59 (3H, s)
**C1'**	169.5	170.3	N/A	N/A
**C2'**	56.2	56.1	3.51 (1H, s)	3.59 (1H, d, J = 2.7 Hz)

Complete ^{1}H and ^{13}C NMR assignments for (−)-halichonic acid B [(−)-**2**]

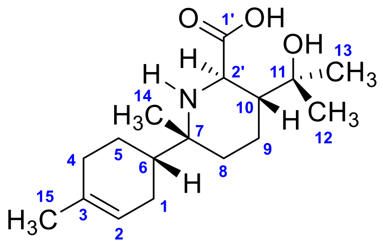

(–)-halichonic acid B

	Reported ^{13}C NMR (150 MHz, CD_3OD)[5]	Observed ^{13}C NMR (100 MHz, CD_3OD)	Reported ^{1}H NMR (600 MHz, CD_3OD)[5]	Observed ^{1}H NMR (400 MHz, CD_3OD)
**C1**	25.5	25.5	1.91 (1H, m)<br>2.03 (1H, m)	1.93 (1H, m)<br>2.02 (1H, m)
**C2**	120.1	120.1	5.43 (1H, br d, J = 4.3 Hz)	5.45 (1H, m)
**C3**	135.4	135.4	N/A	N/A
**C4**	31.6	31.6	2.01 (1H, m)<br>2.10 (1H, m)	2.02 (1H, m)<br>2.12 (1H, m)
**C5**	24.8	24.8	1.29 (1H, m)<br>1.75 (1H, m)	1.30 (1H, m)<br>1.75 (1H, m)
**C6**	36.4	36.3	2.02 (1H, m)	2.04 (1H, m)
**C7**	61.0	61.0	N/A	N/A
**C8**	32.2	32.1	1.61 (1H, m)<br>2.14 (1H, d, *J* = 4.1 Hz)	1.62 (1H, m)<br>2.16 (1H, m)
**C9**	22.2	22.2	1.62 (1H, m)<br>1.86 (1H, m)	1.57 (1H, m)<br>1.86 (1H, m)
**C10**	47.8	47.8	2.02 (1H, m)	2.03 (1H, m)
**C11**	72.6	72.6	N/A	N/A
**C12**	28.9	28.9	1.24 (3H, s)	1.24 (3H, s)
**C13**	25.9	25.9	1.31 (3H, s)	1.31 (3H, s)
**C14**	20.7	20.7	1.26 (3H, s)	1.27 (3H, s)
**C15**	23.3	23.4	1.67 (3H, s)	1.67 (3H, m)
**C1'**	174.2	174.2	N/A	N/A
**C2'**	58.8	58.8	3.59 (1H, d, *J* = 10.1 Hz)	3.58 (1H, d, *J* = 10.3 Hz)

Complete ^{1}H and ^{13}C NMR assignments for (−)-isohalichonic acid B (**11**)

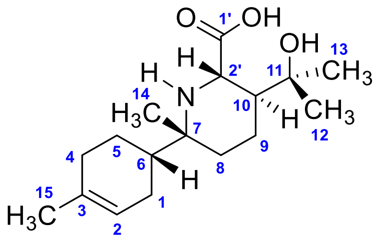

(–)-isohalichonic acid B

	^{13}C NMR (100 MHz, $CDCl_3$)	^{1}H NMR (400 MHz, $CDCl_3$)
**C1**	26.2	2.04 (2H, m)
**C2**	120.1	5.42 (1H, m)
**C3**	135.3	N/A
**C4**	31.6	2.04 (2H, m)
**C5**	24.6	1.30 (1H, m)<br>1.84 (1H, m)
**C6**	44.9	1.89 (1H, m)
**C7**	60.8	N/A
**C8**	33.7	1.62 (1H, m)<br>2.03 (1H, m)
**C9**	23.5	1.64 (1H, m)<br>1.94 (1H, m)
**C10**	49.5	1.87 (1H, m)
**C11**	72.7	N/A
**C12**	29.2	1.24 (3H, s)
**C13**	25.1	1.38 (3H, s)
**C14**	14.5	1.22 (3H, s)
**C15**	23.4	1.67 (3H, m)
**C1'**	175.0	N/A
**C2'**	59.1	3.63 (1H, d, J = 11.3 Hz)

References:

1. Shenvi, R. A.; Pronin, S. V.; Reiher, C. A. *Nature,* **2013,** *501,* 195–199. doi: 10.1038/nature12472
2. Charette, A. B.; Pelletier, G.; Bechara, W. S. *J. Am. Chem. Soc.* **2010,** *132,* 12817–12819. doi: 10.1021/ja105194s
3. Miyaoka, H.; Ota, K.; Hamamoto, Y.; Eda, W.; Tamura, K.; Sawada, A.; Hoshino, A.; Mitome, H.; Kamaike, K. *J. Nat. Prod.,* **2016,** *79,* 996–1004. doi: 10.1021/acs.jnatprod.5b01069
4. Tsukamoto, S.; Raiju, K.; Hitora, Y.; Kato, H.; Ise, Y.; Angkouw, E. D.; Mangindaan, R. E. P. *Tetrahedron Lett.* **2019,** *60,* 1079–1081. doi: 10.1016/j.tetlet.2019.03.030
5. Tsukamoto, S.; Hitora, Y.; Ogura, K.; El-Desoky, A. H. H.; Ise, Y.; Angkouw, E. D.; Mangindaan, R. E. P. *Chem. Pharm. Bull.* **2021,** *69,* 802–805. doi: 10.1248/cpb.c21-00392
